# Achieving Highly Robust Polymeric Microspheres with Efficient and Full‐Color Organic Afterglow for 3D Printing and Anti‐Counterfeiting

**DOI:** 10.1002/advs.202508888

**Published:** 2025-07-29

**Authors:** Pengtao Hu, Yu Lang, Jiahui Sun, Lilin Wang, Zhiling Liang, Zhan Yang, Chunping Ma, Miao Luo, Peiwei Lin, Jiaqing Li, Chunxiong Zheng, Guang Shi, Zhenguo Chi, Bingjia Xu

**Affiliations:** ^1^ School of Chemistry South China Normal University Guangzhou 510006 China; ^2^ School of Environmental and Chemical Engineering Wuyi University Jiangmen 529020 China; ^3^ College of Textile Science and Engineering Wuyi University Jiangmen 529020 China; ^4^ School of Chemistry and Chemical Engineering Key Laboratory of Clean Energy Materials Chemistry of Guangdong Higher Education Institutes Lingnan Normal University Zhanjiang 524048 China

**Keywords:** microsphere, organic afterglow, persistent fluorescence, room‐temperature phosphorescence, thermally activated delayed fluorescence

## Abstract

In this work, highly robust polymer‐based luminescent microspheres with efficient and full‐color organic afterglow are developed for the first time. *N*‐phenylnaphthalen‐1‐amine, *N*‐phenylphenanthren‐9‐amine, and *N*‐phenyltriphenylen‐2‐amine are employed as guest luminogens and embedded into melamine‐formaldehyde polymer matrices with compact and permanent three‐dimensional (3D) covalent networks via host‐guest doping and emulsion polymerization. The resulting polymeric microspheres, with a diameter of ≈2 µm, show blue to yellow‐green organic afterglow at room temperature. Excitingly, their phosphorescence quantum yields and lifetimes are up to 18.0% and 1.39 s, respectively, representing a breakthrough in comprehensive organic afterglow performance for polymer‐based luminescent microspheres. Meanwhile, dual‐mode organic afterglow and persistent red fluorescence with a lifetime of 0.24 s and a quantum yield of 22.2% are also achieved from the microspheres by simply replacing the guest luminogen and the phosphorescence resonance energy transfer approach, respectively. More importantly, the polymer‐based organic afterglow microspheres exhibit outstanding resistance to water and organic solvents. Inspired by the efficient and highly robust organic afterglow, potential applications of the polymeric microspheres are demonstrated in stretchable elastomers, information anti‐counterfeiting, 3D printing, and explosive detection. This work provides a universal strategy for developing polymeric microspheres with efficient, robust, and colorful organic afterglow.

## Graphical abstract

Highly robust polymeric microspheres with efficient ultralong organic phosphorescence, dual‐mode organic afterglow, and persistent fluorescence are developed by embedding different organic luminogens into melamine‐formaldehyde polymer matrices. Their organic afterglow quantum yields and lifetimes are up to 18.0% and 1.39 s under ambient conditions, respectively. The microspheres are applicable for three‐dimensional printing and anti‐counterfeiting.

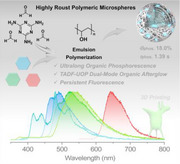

## 1 Introduction

Organic afterglow materials have attracted tremendous attention recently owing to their promising applications in sensing, anti‐counterfeiting, bioimaging, information security, and optoelectronic devices.^[^ ^1^, ^2^, ^3^, ^4^, ^5^, ^6^, ^7^, ^8^, ^9^ ^]^ In most cases, organic afterglow is enabled by ultralong organic phosphorescence (UOP), persistent thermally activated delayed fluorescence (TADF), and long‐lived fluorescence generated by energy transfer from triplet excited states.^[^ ^10^, ^11^, ^12^, ^13^, ^14^ ^]^ Accordingly, several valuable strategies have been developed to facilitate intersystem crossing (ISC) and stabilize triplet excitons, such as incorporation of heteroatoms and heavy halogen atoms, forming *H*‐aggregates, crystal engineering, self‐assembly, polymerization, host‐guest doping, and so forth.^[^ ^15^, ^16^, ^17^, ^18^, ^19^, ^20^, ^21^, ^22^, ^23^ ^]^ In this regard, embedding organic luminogens into polymer matrices via host‐guest doping is more attractive due to the diverse molecular structures and facile synthesis of small organic compounds and the advantages of polymer materials, including flexibility, transparency, low cost, and good processability.^[^ ^24^, ^25^, ^26^, ^27^ ^]^ However, polymer‐based organic afterglow materials usually show unsatisfactory afterglow quantum yields (*Φ*_afterglow_) and lifetimes (*τ*_afterglow_) under ambient conditions.^[^ ^24^, ^28^ ^]^ Moreover, most of them are macroscopic materials in bulk, film, and fiber forms,^[^ ^29^, ^30^, ^31^, ^32^, ^33^, ^34^, ^35^, ^36^, ^37^ ^]^ largely confining their application scope. In this context, it would be nice to miniaturize the polymer‐based organic afterglow materials and confer them with high stability. In this case, these fascinating emitters may be applicable to various scenarios and are apt to combine with diverse materials to construct novel luminescent systems, thereby further unlocking their enormous technological potential.

Polymeric microspheres are spherical or near‐spherical polymer materials with diameters in the range of 0.1–100 µm.^[^ ^38^ ^]^ They have large specific surface areas and excellent biocompatibility and are easily dispersed in various matrices because of the small sizes and regular shapes. As a result, such unique polymer materials, especially those with luminescence characteristics, are widely used in analysis and detection, absorption and separation, recognition and sensing, catalyzed synthesis, and biomedicine. However, polymer‐based luminescent microspheres with organic afterglow properties are very limited (Figure S1, Supporting Information).^[^ ^39^, ^40^ ^]^ Meanwhile, most of them show inferior persistent luminescence performance (**Table** **S1**, Supporting Information),^[^ ^30^, ^39^, ^41^ ^]^ which may be attributed to the large specific surface areas, relatively low ISC efficiencies of the chromophores, and loose structures of the polymer matrices,^[^ ^42^, ^43^, ^44^ ^]^ leading to severe non‐radiative decays of triplet excitons. Moreover, they usually emit blue to green phosphorescence, and those with satisfactory red persistent luminescence have not yet been reported to date.^[^ ^24^, ^45^ ^]^ Consequently, it remains a formidable challenge to develop polymer‐based luminescent microspheres with efficient and full‐color organic afterglow under ambient conditions, especially those that can still produce conspicuous persistent luminescence in different solvents and matrices.

In this work, highly robust polymer‐based luminescent microspheres with efficient and full‐color organic afterglow have been developed for the first time. Herein, naphthalene, phenanthrene, and triphenylene with rigid and conjugated chemical structures,^[^ ^46^, ^47^, ^48^ ^]^ which are favorable for reducing intramolecular motions and tuning phosphorescence emission maximum, have been selected as essential chromophores to construct guest molecules. Meanwhile, phenylamine, which contains a nitrogen atom, is incorporated to the molecules to facilitate the ISC processes.^[^ ^49^, ^50^, ^51^ ^]^ Subsequently, the luminogens are embedded into melamine‐formaldehyde (MF) polymer matrices with compact and permanent 3D covalent networks, which can rigidify the guest molecules and prevent triplet excitons from quenching by oxygen,^[^ ^28^, ^48^, ^52^, ^53^, ^54^ ^]^ via host‐guest doping and emulsion polymerization. It is found that polymer‐based organic afterglow microspheres are successfully achieved, and those doped with the triphenylene derivative show a UOP quantum yield of 18.0% and a lifetime of 1.39 s simultaneously under ambient conditions, representing a state‐of‐the‐art comprehensive organic afterglow performance of polymer‐based luminescent microspheres. Moreover, dual‐mode organic afterglow and persistent red fluorescence have also been achieved from the microspheres by replacing the guest luminogen and the phosphorescence resonance energy transfer (PRET) approach, respectively. In addition, the polymer‐based organic afterglow microspheres exhibit outstanding resistance to water and organic solvents and are apt to be dispersed into different matrices to prepare UOP elastomers, security inks, and 3D printing precursors.

## 2 Results and Discussion

*N*‐phenylnaphthalen‐1‐amine (PNA) is commercially available, while *N*‐phenylphenanthren‐9‐amine (PPA) and *N*‐phenyltriphenylen‐2‐amine (PTA) were prepared through a palladium‐catalyzed carbon‐nitrogen coupling reaction according to the synthetic routes outlined in Scheme S1 (Supporting Information). Their chemical structures and sample purity were characterized by nuclear magnetic resonance spectrometry (^1^H and ^13^C NMR), high‐resolution mass spectrometry (HRMS), and high‐performance liquid chromatography, and satisfactory results were achieved (Figures S2–S15, Supporting Information). As depicted in Figures S16 and S17a (Supporting Information), the emission maxima of PNA, PPA, and PTA in the crystalline state gradually red‐shift with the increase of molecular conjugation and are located at 422, 440, and 492 nm, respectively. The lifetimes of these three compounds were estimated to be 8.73, 7.24, and 3.79 ns (Figure S17b, Supporting Information), identifying their fluorescence attributes. No phosphorescence could be observed from PNA, PPA, and PTA in the solid state at room temperature, which may be ascribed to the remarkable intramolecular motions of the molecules and severe triplet exciton quenching caused by strong π‐π interactions between their polycyclic aromatic hydrocarbon units.^[^ ^55^ ^]^ In addition, PNA, PPA, and PTA show a decreased tendency in fluorescence lifetime, indicating that their intermolecular π‐π interactions likely enhanced along with the enlargement of conjugation of the polycyclic aromatic hydrocarbon moieties.

PNA, PPA, and PTA were subsequently doped into the MF polymers with compact and permanent 3D covalent networks via solution polymerization and hot pressing to prepare polymer films. The resulting PNA‐MF, PPA‐MF, and PTA‐MF films show deep blue emission under the excitation of 365 nm UV light and produce significant yellow‐green, green, and blue persistent luminescence after removing the excitation light source. Evidently, the luminogens can adequately release their phosphorescence potential in the MF polymers under ambient conditions. It is found that the afterglow intensities of the PNA‐MF, PPA‐MF, and PTA‐MF films rise first and then decrease with the elevation of dopant concentrations and reach the maxima at 0.12% in mass ratio (Figures S18–S20, Supporting Information). Similar observations are also recorded for the variations of the afterglow lifetimes. These results may be attributed to the synergistic effect of the increase of luminescence centers and the concentration quenching of triplet excitons. Therefore, PNA‐MF‐0.12%, PPA‐MF‐0.12%, and PTA‐MF‐0.12% were selected as models to further investigate their photophysical properties.

**Figure 1:**
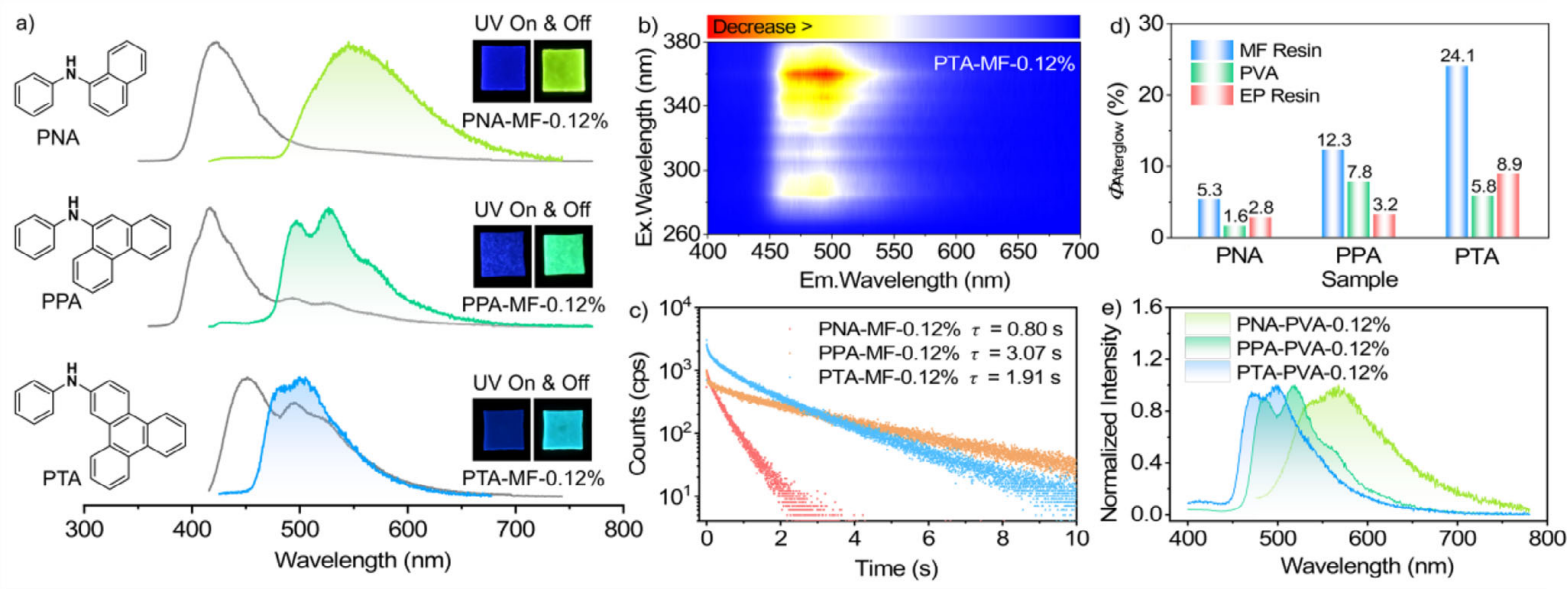
a) Molecular structures of PNA, PPA, and PTA, as well as steady‐state PL spectra and delayed emission spectra of the PNA‐MF‐0.12%, PPA‐MF‐0.12%, and PTA‐MF‐0.12% films under ambient conditions (excitation: 330 nm). Insets are the steady‐state luminescence and UOP images of the doped MF polymer films. b) Variation of the delayed emission spectrum of PTA‐MF‐0.12% under different excitations. c) Emission decay curves and fitting lifetimes of the PNA‐MF‐0.12%, PPA‐MF‐0.12%, and PTA‐MF‐0.12% films under ambient conditions. d) Afterglow quantum yields of PNA, PPA, and PTA in different polymer matrices under ambient conditions. e) Delayed emission spectra of the PNA‐PVA‐0.12%, PPA‐PVA‐0.12%, and PTA‐PVA‐0.12% films under ambient conditions.

As shown in **Figure** **1**a, PNA, PPA, and PTA in the MF polymer films exhibit a fluorescence emission maximum at 406, 400, and 436 nm, while their phosphorescence bands are located at yellow‐green (*λ*_phos._ = 540 nm), green (*λ*_phos._ = 486, 518 nm), and blue (*λ*_phos._ = 472, 495 nm) light regions, respectively. In the meantime, the phosphorescence spectra of the samples are unchanged with the variation of excitation wavelength (Figure 1b; Figure S21, Supporting Information), suggesting that the molecules are dispersed well in the matrices, and the observed room‐temperature phosphorescence probably originates from single molecules of the luminogens.^[^ ^4^, ^56^, ^57^ ^]^ After ceasing the UV light excitation, the doped polymer films are capable of generating intense organic afterglow with a duration of up to 30 s (Figure S22 and Video S1, Supporting Information). The fitting results of emission decay curves indicate that PNA‐MF‐0.12%, PPA‐MF‐0.12%, and PTA‐MF‐0.12% show a phosphorescence lifetime (*τ*_phos._) of 0.80, 3.07, and 1.91 s under ambient conditions (Figure 1c), respectively. Moreover, their phosphorescence quantum yields (*Φ*_phos._) are estimated to be 5.3%, 12.3%, and 24.1% (Figure 1d). By contrast, the *τ*_phos._ and *Φ*_phos._ values of the blank MF polymer film (*λ*_PL_ = 371 nm, *λ*_phos._ = 498 nm) are only 430 ms and 0.65%, respectively, indicating little influence on the persistent luminescence performance of the doped samples (Figure S23, Supporting Information). It is noteworthy that PTA‐MF‐0.12% shows a high *Φ*_phos._ value of 24.1% and an ultralong lifetime of 1.91 s  , which are superior to most of the reported polymer‐based organic afterglow materials.^[^ ^12^, ^24^, ^34^ ^]^ Intriguingly, PNA, PPA, and PTA display similar phosphorescent spectra when the polymer matrices are replaced by polyvinyl alcohol (PVA) and epoxy (EP) resin (Figure 1e; Figure S24, Supporting Information). However, their *τ*_phos._ and/or *Φ*_phos._ were significantly reduced (Figure 1d; Figure S25, Supporting Information). In addition, the UOP intensities and lifetimes of the doped PVA and EP polymer films in air are apparently reduced in comparison with those under vacuum (Figures S26–S31, Supporting Information). By contrast, the atmosphere has little influence on the UOP performance of PNA‐MF‐0.12%, PPA‐MF‐0.12%, and PTA‐MF‐0.12%. These results further verify that the MF polymers may render more rigid and compact microenvironments, effectively suppressing the intramolecular motions of the guest luminogens and isolating the oxygen in air.^[^ ^52^, ^54^, ^58^ ^]^

**Figure 2:**
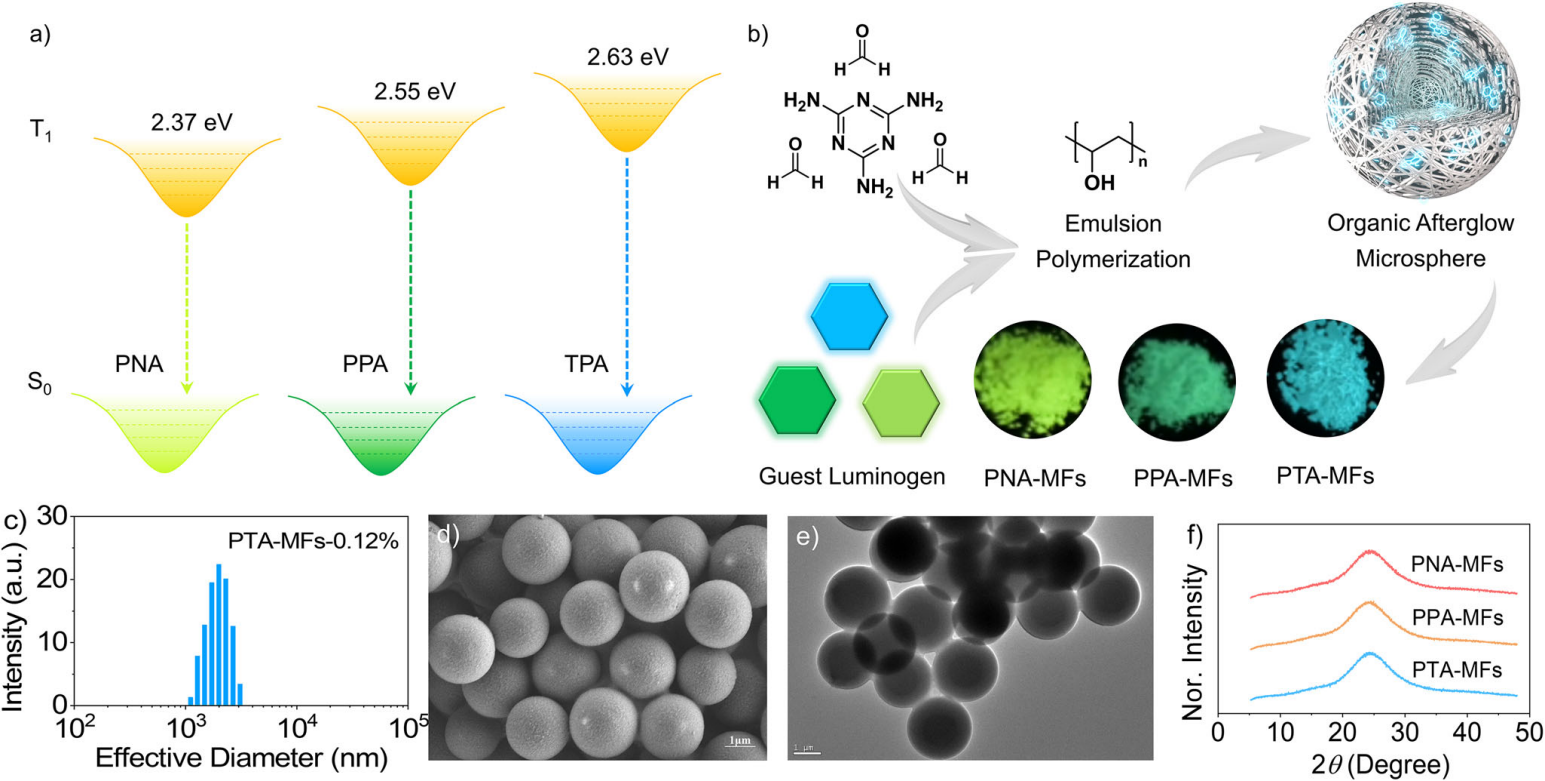
a) T_1_ energy levels of PNA, PPA, and TPA obtained from theoretical calculations. b) Schematic diagram for the preparation of the polymeric microspheres with organic afterglow properties and the UOP images of the PNA‐MFs, PPA‐MFs, and PTA‐MFs microspheres. c) Effective diameter distribution of the PTA‐MFs‐0.12% microspheres dispersed in water. d) Scanning electron microscope image of the PTA‐MFs‐0.12% microspheres (scale bar: 1 µm). e) Transmission electron microscope image of the PTA‐MFs‐0.12% microspheres (scale bar: 1 µm). f) X‐ray diffraction patterns of the PNA‐MFs, PPA‐MFs, and PTA‐MFs microspheres.

Given the excellent phosphorescence performance and tunable lowest triplet excited state (T_1_) energy levels, PNA, PPA, and PTA were embedded into the MF polymer matrices to prepare polymeric microspheres with organic afterglow properties through host‐guest doping and emulsion polymerization (**Figure** **2**a,b). As depicted in Figure 2c and Figure S32 (Supporting Information), the resulting solid granules PNA‐MFs‐0.12%, PPA‐MFs‐0.12%, and PTA‐MFs‐0.12% show an effective diameter of 1.97, 2.03, and 2.10 µm, respectively. In the meantime, their polydispersity indexes (PDI) reach 0.83, 0.88, and 0.87. Figure 2d and Figure S33 (Supporting Information) give the scanning electron microscope images of the samples. PNA‐MFs‐0.12%, PPA‐MFs‐0.12%, and PTA‐MFs‐0.12% exhibit spherical morphologies in micrometer scale (≈2 µm) with smooth surfaces. Moreover, the observation from transmission electron microscope indicates that they are solid microspheres (Figure 2e). In addition, no sharp and intense diffraction peaks are recorded in the X‐ray diffraction patterns, suggesting the microspheres are amorphous (Figure 2f). These results unambiguously demonstrate that doped polymeric microspheres with 3D covalent networks are successfully achieved.

**Figure 3:**
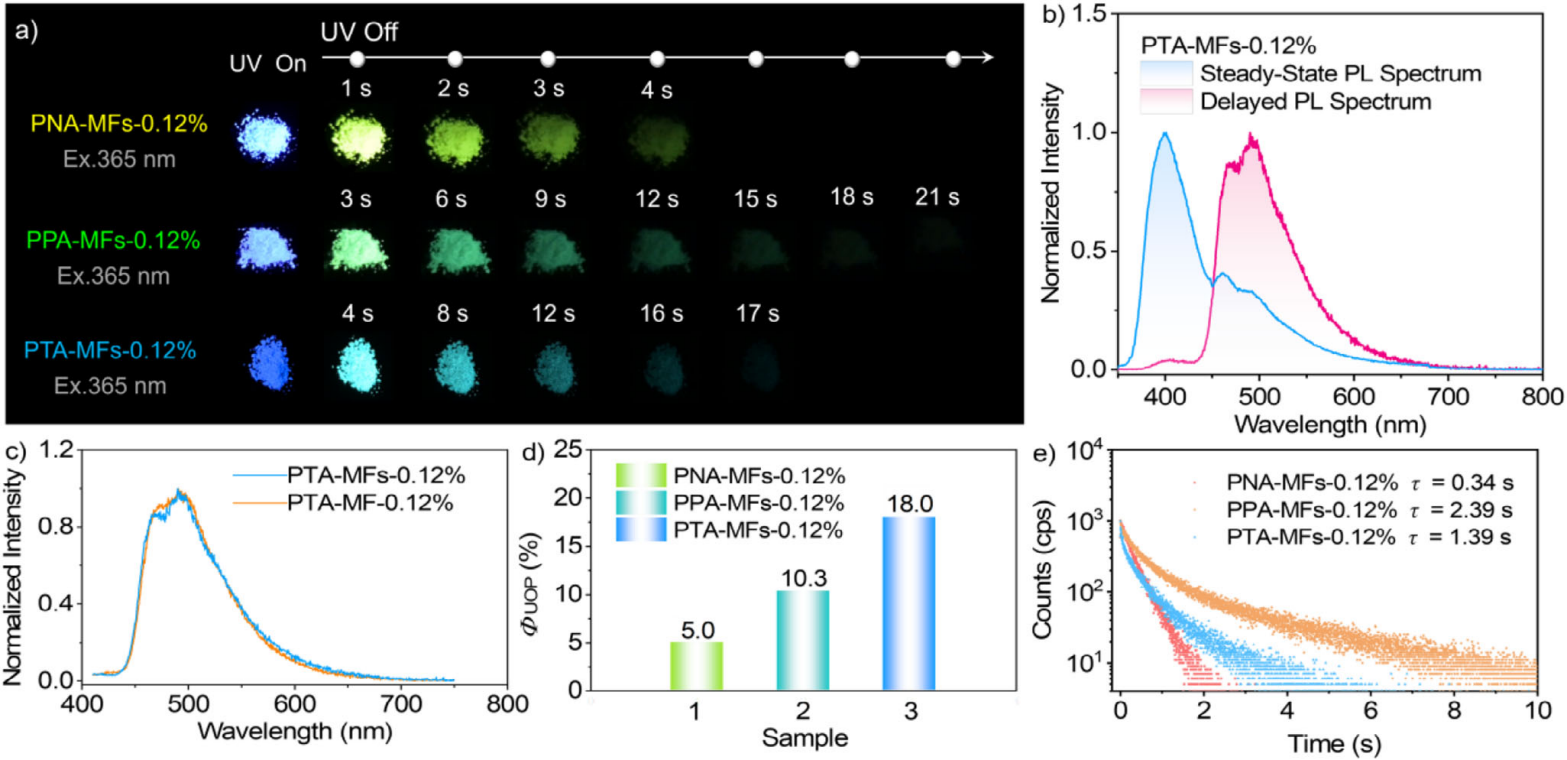
a) Steady‐state luminescence and UOP images of the doped polymeric microspheres under ambient conditions (light density: 30 mW cm^−2^). b) Steady‐state PL and delayed emission spectra of the PTA‐MFs‐0.12% microspheres under ambient conditions (excitation: 330 nm). c) Delayed emission spectra of the PTA‐MFs‐0.12% microspheres and PTA‐MF‐0.12% polymer film at room temperature. d) *Φ*_phos._ values of the doped polymeric microspheres under ambient conditions. e) Emission decay curves and fitting lifetimes of the doped polymeric microspheres under ambient conditions.

Notably, PNA‐MFs‐0.12%, PPA‐MFs‐0.12%, and PTA‐MFs‐0.12% can produce bright UOP with a duration of up to 21 s at room temperature upon the excitation of 365 nm UV light (**Figure** **3**a; Video S2, Supporting Information). Their persistent luminescence colors are yellow‐green, green, and blue, respectively. Accordingly, it is facile to tune the afterglow emissions of the polymeric microspheres by varying the conjugations of phosphorescent chromophores in the guest luminogens. It is found that PNA, PPA, and PTA in the polymeric microspheres show a UOP maximum at 540, 525, and 492 nm (Figure 3b; Figure S34, Supporting Information), respectively, which are in accordance with those of the doped MF polymer films (Figure 3c; Figure S35, Supporting Information). Excitingly, the *Φ*_phos._ of PNA‐MFs‐0.12%, PPA‐MFs‐0.12%, and PTA‐MFs‐0.12% under ambient conditions are estimated to be 5.0%, 10.3%, and 18.0% (Figure 3d). In the meantime, their *τ*_phos._ values reach 0.34, 2.39, and 1.39 s (Figure 3e), respectively, which are even superior to most of the polymer‐based organic afterglow materials in bulk, film, and fiber forms. To the best of our knowledge, polymeric microspheres with *Φ*_phos._ over 10% and *τ*_phos._ over 1.0 s simultaneously have not yet been reported to date.^[^ ^24^, ^34^, ^39^ ^]^ As compared to PNA‐MFs‐0.12%, PPA‐MFs‐0.12% and PTA‐MFs‐0.12% show much higher *Φ*_phos._ and *τ*_phos._ values, which may be associated with the elevation of ISC efficiency and reduction of intramolecular motion of the guest molecules.^[^ ^59^ ^]^ After microspheroidization, these three organic luminogens, especially PPA and PTA, still present prominent UOP performance in the MF polymer matrices, fully demonstrating that the molecular design strategy and material preparation approach are useful for achieving excellent polymer‐based organic afterglow microspheres. Besides, the *Φ*_phos._, *τ*_phos._, and persistent luminescence colors of the polymeric microspheres can be easily tuned by simply varying the chemical structures of the guest luminogens.

**Figure 4:**
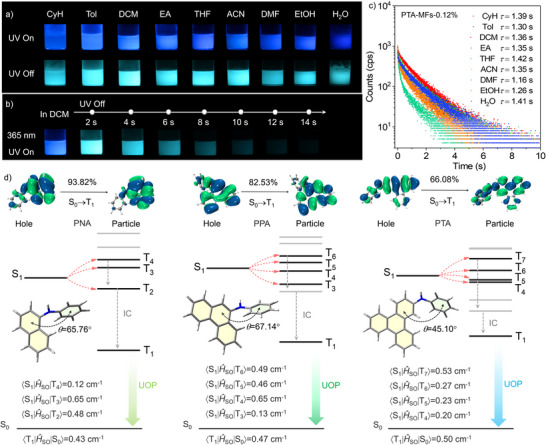
a) Steady‐state luminescence and UOP images of the PTA‐MFs‐0.12% microspheres in water and different organic solvents under ambient conditions. b) Steady‐state luminescence and afterglow images of the PTA‐MFs‐0.12% microspheres in DCM under ambient conditions (light density: 30 mW cm^−2^). c) Emission decay curves and fitting lifetimes of the PTA‐MFs‐0.12% microspheres after immersion in water and different organic solvents for 10 days under ambient conditions. d) NTO characteristics of the T_1_ excited states, optimized molecular conformations, S_1_ and T_n_ energy levels, and SOC constants of PNA, PPA, and PTA. *θ* represents the dihedral angle between the two aromatic groups of the molecule.

Subsequently, PTA‐MFs‐0.12% was selected as the representative to evaluate the solvent resistance of the doped polymeric microspheres, which is of great importance to their real‐world applications. As depicted in **Figure** **4**a and Video S3 (Supporting Information), the PTA‐MFs‐0.12% microspheres can emit deep‐blue emission under the illumination of 365 nm UV light and produce bright blue UOP after removing the excitation light source in water and common organic solvents, including cyclohexane (CyH), toluene (Tol), dichloromethane (DCM), ethyl acetate (EA), tetrahydrofuran (THF), acetonitrile (CAN), *N*,*N*’‐dimethylformamide (DMF), and ethanol (EtOH). Impressively, they are capable of generating significant organic afterglow with a duration of 14 s, even in dichloromethane (Figure 4b), which is an organic solvent with high dissolving and infiltration capacities. Moreover, after immersion in water and different organic solvents for 10 days, PTA‐MFs‐0.12% still produce apparent persistent luminescence, and their UOP lifetime shows little change (Figure 4c). These findings indicate that the polymeric microspheres possess compact and permanent 3D covalent networks, which perfectly protect the phosphorescent molecules in the matrices, thereby enabling highly robust organic afterglow under ambient conditions. It is noteworthy that polymer‐based luminescent microspheres with excellent organic afterglow performance and outstanding resistance to water and organic solvents have not been reported so far.

Theoretical calculations were subsequently conducted to gain an in‐depth insight into the observed organic afterglow properties. As depicted in Figure 4d, PNA, PPA, and PTA show twisted molecular conformations, with a large dihedral angle (*θ*) of 65.76, 67.14, and 45.10° between the involving phenyl ring and polycyclic aromatic hydrocarbon moiety, respectively. It is found that their possible electron transition pathways are HOMO‐2→LUMO+1 (*f* = 0.139), HOMO‐2→LUMO+1 (*f* = 0.538), and HOMO‐1→LUMO (*f* = 0.418) (Figure S36, Supporting Information), and nπ* transitions occur in the S_0_→S_1_ transition processes of the luminogens (Figure S37, Supporting Information). PTA exhibits four possible ISC channels (S_1_→T_4_, T_5_, T_6_, T_7_) with large spin‐orbit coupling constants (〈S_1_|*Ĥ*_SO_|T_n_〉) of 0.20, 0.23, 0.27, and 0.53 cm^−2^, suggesting its high ISC efficiency, which is favorable for generating substantial triplet excitons. In the meantime, its 〈T_1_|*Ĥ*_SO_|S_0_〉 reaches 0.50 cm^−2^, partially allowing the T_1_→S_0_ electron transition process. It is noteworthy that intramolecular motions of the luminogen are probably reduced due to the bulk and rigid triphenylene unit. Moreover, the compact 3D covalent network of the MF polymer would also largely restrain the intramolecular motions of the guest molecules, thereby stabilizing the triplet excitons and preventing them from oxygen quenching. In this case, the non‐radiative decays of the triplet excitons are probably suppressed significantly. As a result, PTA exhibits conspicuous organic afterglow with an ultralong lifetime and a large *Φ*_phos._ value in the MF polymer matrix and exhibits excellent persistent luminescence performance even in the polymeric microspheres under ambient conditions. PPA likewise has four possible ISC channels (S_1_→T_4_, T_5_, T_6_, T_7_), with large 〈S_1_|*Ĥ*_SO_|T_n_〉 (n = 3, 4, 5, 6) values in the range of 0.13–0.65 cm^−2^. Nevertheless, in the case of PNA, the ISC channels from S_1_ to triplet excited states (S_1_→T_2_, T_3_, T_4_) reduce. As compared to those of PPA and PNA, the 〈T_1_|*Ĥ*_SO_|S_0_〉 value of PTA slightly increases. Meanwhile, the sizes and rigidities of the essential phosphorescent chromophores of the guest luminogens gradually elevate from PNA to PPA and then to PTA, which likely result in a decline in molecular motion and thereby restrain the non‐radiative decay. Consequently, the *Φ*_phos._ of PTA is significantly higher than that of PPA in both the MF polymer film and polymeric microsphere, and PNA shows the lowest *Φ*_phos._ under ambient conditions. Notably, PPA has a large T_2_−T_1_ energy gap (0.658 eV), which may lead to a relatively slow internal conversion (IC) process. Furthermore, it exhibits higher molecular rigidity in comparison with PNA but a smaller 〈T_1_|*Ĥ*_SO_|S_0_〉 value as compared to PTA. For these reasons, PPA displays a longer UOP lifetime than the other two luminogens. In addition, the T_1_ excited states of PNA, PPA, and PTA are mainly characterized by a ^3^ππ configuration. In this context, the UOP of the luminogens probably originates from ^3^LE state transitions of the long‐lived triplet excitons, which are in accordance with the experimental results.

**Figure 5:**
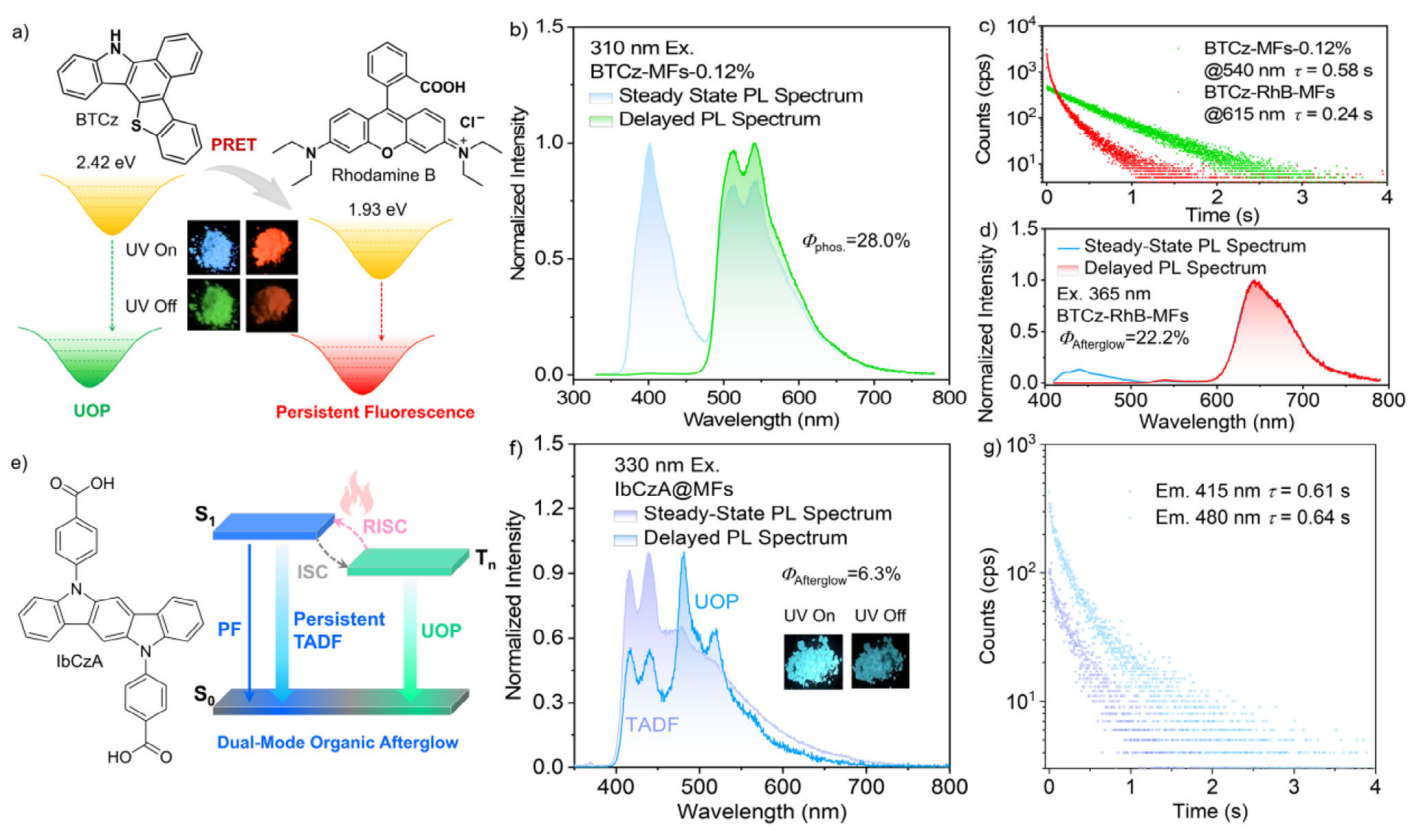
a) Molecular structures and T_1_ energy levels of BTCz and Rhodamine B as well as the phosphorescence resonance energy transfer (PRET) between these two luminogens. Insets are the steady‐state luminescence and afterglow images of the BTCz‐MFs‐0.12% and BTCz‐RhB‐MFs. b) Steady‐state PL and delayed emission spectra of the BTCz‐MFs‐0.12% under ambient conditions. c) Emission decay curves and fitting lifetimes of the BTCz‐MFs‐0.12% and BTCz‐RhB‐MFs under ambient conditions. d) Steady‐state PL and delayed emission spectra of the BTCz‐MFs‐0.12% and BTCz‐RhB‐MFs under ambient conditions. e) Molecular structure and schematic diagram of the dual‐mode organic afterglow of IbCzA in the MF polymeric microspheres. f) Steady‐state PL and delayed emission spectra of the IbCzA‐MFs‐0.12% under ambient conditions. g) Emission decay curves and fitting lifetimes of the IbCzA‐MFs‐0.12% microspheres under ambient conditions.

To verify the universality of the organic afterglow polymeric microsphere preparation strategy, a reported phosphorescent luminophore BTCz was employed to replace PNA, PPA, and PTA as the guest molecule. Similarly, the resulting polymeric microsphere BTCz‐MFs‐0.12% (*d* = 1.92 µm, PDI = 0.85) (Figure S38, Supporting Information) can likewise produce intense persistent luminescence under ambient conditions (**Figure** **5**a; Video S2, Supporting Information). It shows a green organic afterglow, with phosphorescence peaks at around 513 and 540 nm (Figure 5b). In the meantime, the *Φ*_phos._ and *τ*_phos._ of BTCz‐MFs‐0.12% are up to 28.0% and 0.58 s (Figure 5c), respectively, which are even comparable to those of the BTCz‐doped PVA film.^[^ ^51^ ^]^ Further investigation reveals that the phosphorescence emission band of BTCz has a large overlap with the absorption spectrum of Rhodamine B (Figure S39, Supporting Information), which is a classic red‐fluorescence dye. Given this, BTCz (0.12%) and Rhodamine B were co‐doped into the MF polymeric microspheres to achieve persistent red fluorescence via PRET. Excitingly, when the doping concentration of Rhodamine B reaches 0.60% in mass ratio, the polymeric microsphere BTCz‐RhB‐MFs (*d* = 1.98 µm, PDI = 0.89) produces a highly bright red organic afterglow. In its delayed emission spectrum (Figure 5d), only a weak emission band is recorded at around 540 nm, which originates from the phosphorescence of BTCz. Meanwhile, the *τ*_phos._ of BTCz significantly reduces, from 0.58 s to 61.94 ms (Figure S40, Supporting Information). In sharp contrast, an intense red emission band that is in accordance with the fluorescence of Rhodamine B emerges at 615 nm. Moreover, no organic afterglow is observed from the polymeric microspheres doped with Rhodamine B (Figure S41, Supporting Information). These observations indicate that PRET from BTCz to Rhodamine B occurs in the co‐doped polymeric microspheres. Notably, the energy transfer efficiency of such a fascinating persistent luminescence system reaches 89.3%, thus resulting in a high persistent fluorescence quantum yield (*Φ*_afterglow_ = 22.2%) and an ultralong lifetime (*τ*_afterglow_ = 0.24 s) of the BTCz‐RhB‐MFs. Accordingly, the afterglow color of the doped MF polymeric microspheres is successfully extended to the red region, and satisfactory red persistent luminescence is also achieved under ambient conditions through the strategy of combining host‐guest doping, PRET, and emulsion polymerization.

Recently, IbCzA has been identified as an organic luminogen with dual‐mode organic afterglow properties (Figure 5e).^[^ ^14^ ^]^ Given this, it was also employed as the guest molecule and embedded into the MF polymeric microsphere using the same procedures. As depicted in Figures 5f and Figure S42 (Supporting Information), the resulting microsphere IbCz‐ MFs‐0.12% (*d* = 2.04 µm, PDI = 0.87) produces a significant blue afterglow (*Φ*_afterglow_ = 6.3%) under ambient conditions after removing the excitation light source. Its delayed luminescence presents two emission bands. The former one is in line with the fluorescent emission profile, which can be attributed to TADF, manifesting by the variation trends in delayed emission intensity and emission decays under different temperatures (Figure S43, Supporting Information). The latter one shows two emission peaks at 480 and 518 nm, originating from UOP. Meanwhile, these two emission bands exhibit similar lifetimes, which are estimated to be 0.61 and 0.64 s (Figure 5g), respectively. These findings suggest that IbCz‐MFs‐0.12% is capable of generating dual‐mode organic afterglow composed of persistent TADF and UOP. Evidently, it is a universal strategy to produce polymeric microspheres with excellent organic afterglow performance by embedding organic luminogens into the MF polymer matrices with compact and permanent 3D covalent networks via the combination of host‐guest doping and emulsion polymerization. Upon such a useful strategy, polymeric microspheres with full‐color organic afterglow and highly robust UOP, persistent fluorescence, and dual‐mode persistent luminescence have been successfully developed for the first time.

**Figure 6:**
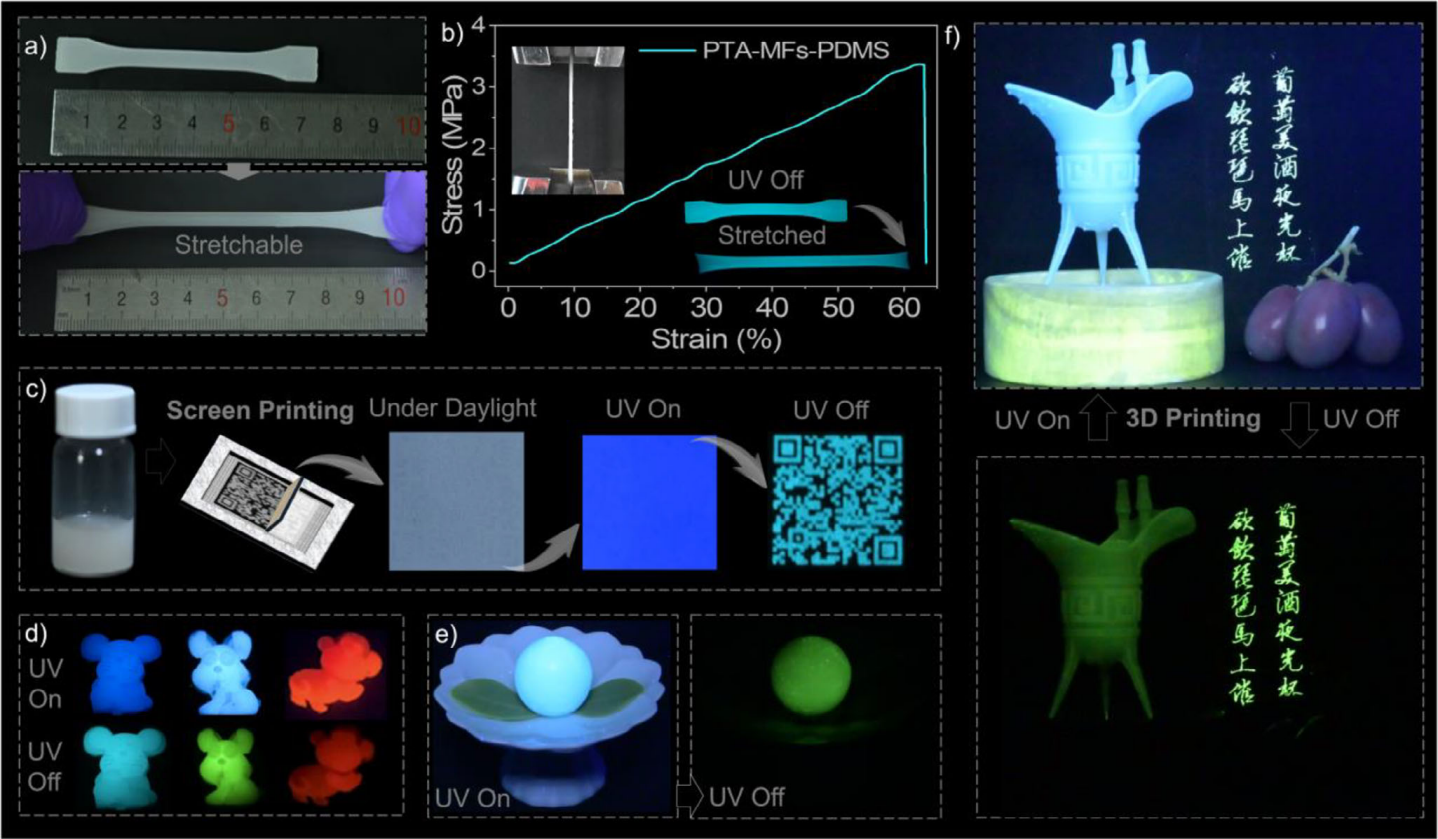
a) Images of the PDMS strip containing PTA‐MFs‐0.12% microspheres before and after stretching. b) Stress‐strain curve of the PDMS strip containing PTA‐MFs‐0.12% microspheres. Insets are the image of the tensile test and the UOP images of the PDMS strip before and after stretching. c) A security ink prepared by employing acrylic acid resin as the matrix and PTA‐MFs‐0.12% microspheres as the pigments and its application in the preparation of a quick response code with organic afterglow properties for anti‐counterfeiting. d) Steady‐state PL and afterglow images of the mouse, rabbit, and monkey models prepared by dispersing the PTA‐MFs‐0.12%, PPA‐MFs‐0.12%, and BTCz‐RhB‐MFs microspheres in ethylene‐vinyl acetate copolymer, respectively. e) Steady‐state PL and afterglow images of a peach prepared by 3D printing. f) Steady‐state PL and afterglow images of a Chinese ancient wine goblet and a Chinese poem prepared by 3D printing and screen printing, respectively.

Inspired by the efficient and highly robust organic afterglow, the doped MF polymeric microspheres were composited with some other polymers to explore their application potential. Owing to the small size and spherical morphology, PTA‐MFs‐0.12% can be easily dispersed in polydimethylsiloxane (PDMS) and has a negligible influence on the mechanical property of the elastomer (**Figure** **6**a). As demonstrated by the stress‐strain measurement result, the luminescent elastomer PTA‐MFs‐0.12%/PDMS presents excellent stretchability and shows a satisfactory elongation at break (>60%) (Figure 6b). Furthermore, it can produce homogeneous and conspicuous blue UOP under ambient conditions, serving as an ideal candidate to construct stretchable organic afterglow systems. Figure 6c depicts a luminescent security ink that is prepared by simply mixing the PTA‐MFs‐0.12% microspheres with a commercially available acrylic acid resin, which is one of the most commonly used ink matrices. It is found that a transparent quick response (QR) code, which is difficult to observe with the naked eye under daylight, can be easily printed on white paper using the security ink through screen printing. Under the excitation of 365 nm UV light, the resulting QR code produces a deep blue emission similar to the fluorescence background of the paper. Thus, it cannot be recognized by scanning equipment. However, the hidden QR code emerges via the blue organic afterglow of the PTA‐MFs‐0.12% microspheres after removing the excitation light source and maintains for over 10 s. In this case, the encrypted information can be deciphered by scanning the QR code using a mobile phone. These results suggest that the doped polymeric microspheres with organic afterglow properties are promising for information anti‐counterfeiting applications.

Figure 6d demonstrates the mouse, rabbit, and monkey models that are prepared by filling the PTA‐MFs‐0.12%, PPA‐MFs‐0.12%, and BTCz‐RhB‐MFs microspheres into the ethylene‐vinyl acetate copolymer matrix, respectively. Upon the excitation of UV light, the 3D elastic models exhibit blue, green, and red persistent luminescence, suggesting their potential applications in advertising and decoration. More impressively, the persistent luminescent polymeric microspheres are also applicable for the construction of anti‐counterfeiting systems through 3D printing by mixing with a commercially available light‐cured resin. Following this approach, a luminescent peach is achieved by employing BTCz‐MFs‐0.12% as the organic afterglow additive (Figure 6e). Its emission color turns from blue to green after ceasing the UV light excitation, accompanied by the vanishment of the background, thus allowing the identification of the genuine product. Figure 6f demonstrates a Chinese ancient wine goblet and a famous Chinese poem, which are prepared by 3D printing and screen printing using BTCz‐MFs‐0.12% as the afterglow additive and light‐cured resin and acrylic acid resin as the matrices, respectively. Under the excitation of 365 nm UV light, the goblet and poem show blue emission and thereby enable a poetic scene with the surroundings. After removing the excitation light source, they are still clearly observed via the green UOP of the BTCz‐MFs‐0.12% microspheres (Video S4, Supporting Information), reappearing the beautiful picture of “grape wine and luminous goblet”. These results indicate that the afterglow polymeric microspheres are apt to composite with various matrices to prepare new organic afterglow materials and may have important applications in stretchable persistent luminescent devices, security ink, 3D/4D printing, information encryption, and multi‐level anti‐counterfeiting. In addition, PTA‐MFs‐0.12% was used for explosive detection. As shown in Figure S44 (Supporting Information), upon exposure to a trinitrophenol (TNP) solution with a concentration of 50 µM, the UOP intensity of the microspheres decreased from 1.57 × 10^4^ to 1.11 × 10^3^ a.u. Even in the TNP solution with a concentration of 1 µm, a significant reduction in UOP intensity was observed. These results suggest the potential of the organic afterglow microspheres for detecting traces of explosives. The UOP quenching in PTA‐MFs‐0.12% is likely attributable to electron transfer from the LUMO of the guest molecules to the LUMO of TNP (Figure S45, Supporting Information), followed by non‐radiative deactivation back to the ground state.

## 3 Conclusion

In summary, highly robust polymeric microspheres with efficient and full‐color organic afterglow have been developed for the first time by embedding organic luminogens into the MF polymer matrices with a compact and permanent 3D covalent network via host‐guest doping and emulsion polymerization. PTA‐MFs‐0.12% shows a conspicuous organic afterglow with a high *Φ*_phos._ of 18.0% and an ultralong *τ*_phos._ of 1.39 s under ambient conditions, representing state‐of‐the‐art comprehensive organic afterglow performance of luminescent polymeric microspheres. In the meantime, the doped MF polymeric microspheres can still produce significant persistent luminescence and exhibit little change in UOP lifetimes after immersion in water and various organic solvents for over 10 days, demonstrating outstanding resistance to water and organic solvents. By employing BTCz and Rhodamine B as the guest molecules, the organic afterglow color of the polymeric microsphere is successfully extended to the red region via PRET. Moreover, dual‐mode organic afterglow composed of persistent TADF and UOP is also achieved from polymeric microspheres using IbCzA as the guest luminogen. Inspired by the excellent organic persistent luminescence performance and outstanding stability, the polymeric microspheres are composited with different polymer matrices to demonstrate their potential applications in stretchable elastomers, information anti‐counterfeiting, and 3D printing, and are directly used for explosive detection. This work presents a universal strategy for developing efficient and highly robust polymeric microspheres with colorful organic afterglow under ambient conditions, which may facilitate innovative applications of organic afterglow materials in flexible and stretchable optoelectronic devices, information security, detection, and advanced printing technology.

## Conflict of Interest

The authors declare no conflict of interest.

## Supporting information



Supporting Information

Supplemental Video 1

Supplemental Video 2

Supplemental Video 3

Supplemental Video 4

## Data Availability

The data that support the findings of this study are available in the supplementary material of this article.
