# Supplementary material for: Achieving Highly Robust Polymeric Microspheres with Efficient and Full‐Color Organic Afterglow for 3D Printing and Anti‐Counterfeiting
Source: Adv Sci (Weinh). 2025 Jul 29;12(40):e08888. doi: 10.1002/advs.202508888 (PMC12561192; doi:10.1002/advs.202508888)
Supplement: Supplementary file 1 — Supporting Information [file ADVS-12-e08888-s002.pdf]

# Supporting Information

## Achieving Highly Roust Polymeric Microspheres with Efficient and Full-Color Organic Afterglow for 3D Printing and Anti-Counterfeiting

*Pengtao Hu, Yu Lang, Jiahui Sun, Lilin Wang, Zhiling Liang, Zhan Yang, Chunping Ma,* Miao Luo, Peiwei Lin, Jiaqing Li, Chunxiong Zheng, Guang Shi,* Zhenguo Chi, and Bingjia Xu**

**Contents**

I. Experimental section .......... 2
Chemical reagents and materials .......... 2
Instruments and Measurements .......... 2
Theoretical calculations .......... 2
Synthesis .......... 3
II. Typical Luminescent Microspheres with Organic Afterglow Properties .......... 7
III. $^1$H NMR, $^{13}$C NMR, and High-Resolution Mass Spectra Results .......... 8
IV. Photophysical Properties of the Luminophores and the Doped MF Polymer Films .......... 15
V. Photophysical Properties of the Doped MF Polymeric Microspheres .......... 21
References .......... 30

# I. Experimental section

## Chemical reagents and materials

Tris(dibenzylideneacetone) dipalladium was purchased from *Aladdin*. Sodium *tert*-butoxide, 2-dicyclohexylphosphino-2',4',6'-triisopropylbiphenyl, triethylamine, aniline, 1-aminopyrene, 9-bromophenanthrene, and 2-bromotriphenylene were purchased from *Energy Chemical.* Rhodamine B was purchased from *Macklin*. PNA and BTCz were purchased from *Bidepharm* and purified by silica gel column chromatography prior to use. IbCzA was prepared according to the literature method reported by our group. Other reagents and organic solvents were purchased from Guangzhou *Zeyuan* Company (China) with analytical grade and used without further purification.

## Instruments and Measurements

$^{1}$H and $^{13}$C nuclear magnetic resonance spectra of the intermediates and final products were obtained on a Bruker AVANCE spectrometer (600 MHz) by employing DMSO-$d_6$ and $CDCl_3$ as the solvent and tetramethylsilane as the internal standard. High-resolution mass spectrometry was carried out on the instrument of LTQ Orbitrap LCMS (LTQ Orbitrap Elite) to determine the exact molecular weights of the compounds. Wide-angle XRD patterns were recorded at 297 K by a Bruker X-ray diffractometer (D8 ADVANCE, Germany) at a 4° (2θ)/min scan rate. In addition, steady-state PL spectra, delayed emission spectra, absolute PL quantum yields, and time-resolved emission decay curves were collected using a spectrometer (FLS980) equipped with a calibrated integrating sphere and a thermostat (Oxford) from Edinburgh Instruments. High-performance liquid chromatography results were obtained from SHIMADZU LC-20A using isopropanol/*n*-hexane as eluent. A 365 nm UV lamp with a power of 40 W was used for evaluating the afterglow duration of the samples.

## Theoretical calculations

The molecular geometries of PNA, PPA, PTA and TNP at ground state were optimized using the density functional theory (DFT) method at the B3LYP/6-311g (d, p) level in the Gaussian 09 program.[1] The energy levels of the singlet ($S_n$) and triplet excited states ($T_n$) were estimated through a combination of TD-DFT and M062X at the def2-TZVP level. Herein, the possible $T_n$ states are considered to lie within the range of $E_{S1} \pm 0.30$ eV.[2] Spin-orbit coupling (SOC) matrix elements between the singlet and triplet excited states were calculated through the ORCA 5.0.3 software based on the TD-DFT results.[3] Natural transition orbital (NTO) analysis

was performed based on the TD-DFT data, and the results were extracted through the Multiwfn (version 3.8) software and subsequently visualized via the VMD (version 1.9.3) software.[4]

**Synthesis**

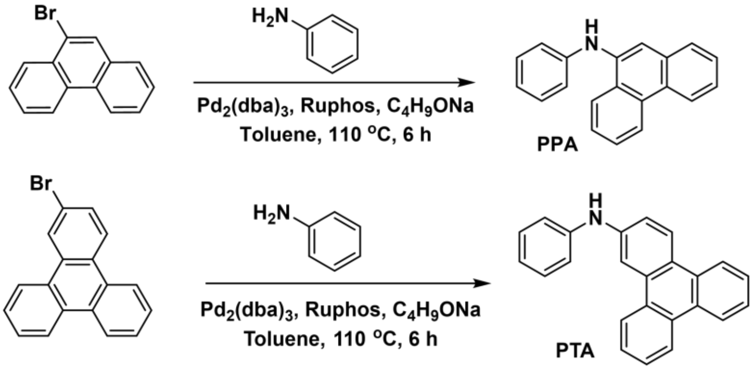


**Scheme S1.** The synthetic routes of PPA and PTA.

***N-phenylnaphthalen-1-amine (PNA)*** Commercially available PNA was further purified by silica gel column chromatography using dichloromethane/petroleum ether (v/v = 1:9) as the eluent prior to use. $^1$H NMR (600 MHz, DMSO-$d_6$) δ 8.22–8.18 (s, 1H), 8.18–8.15 (d, $J$=8.3 Hz, 1H), 7.92-7.86 (m, 1H), 7.55–7.47 (m, 3H), 7.42–7.38 (t, $J$=7.8 Hz, 1H), 7.34–7.30 (d, $J$=7.4 Hz, 1H), 7.25–7.19 (t, $J$=7.8 Hz, 2H), 7.07–7.02 (t, $J$=7.6 Hz, 2H), 6.84–6.79 (t, $J$=7.3 Hz, 1H). HPLC: purity 99.9%.

***Synthesis of N-phenylphenanthren-9-amine (PPA)*** 9-Bromophenanthrene (0.50 g, 1.94 mmol), aniline (0.27 g, 2.92 mmol), 2-dicyclohexylphosphino-2',4',6'-triisopropylbiphenyl (80 mg), sodium *tert*-butoxide (0.56 g, 5.83 mmol), and toluene (30 mL) were added to a three-necked flask. The reaction mixture was degassed by bubbling with argon for about 30 min. Subsequently, $Pd_2(dba)_3$ (0.05 g, 0.09 mmol) was added, and the reaction mixture was stirred at 110 °C under an argon atmosphere for 6 h. After cooling to room temperature, the mixture was filtered, and the solvent of the filtrate was removed by rotary evaporation. The crude product was then separated and purified by silica gel column chromatography with dichloromethane and petroleum ether (v/v=1:4) as eluent. The resulting solid was recrystallized twice from dichloromethane/methanol under ultrasonication to give a white solid powder (0.35 g, yield 66.8%). $^1$H NMR (600 MHz, DMSO-$d_6$) δ 8.88–8.84 (d, $J$ = 8.2 Hz, 1H), 8.76–8.70 (d, $J$ = 8.0 Hz, 1H), 8.33–8.28 (d, $J$ = 8.1, 1H), 8.27–8.18 (s, 1H), 7.81–7.76 (m, 1H), 7.76–7.71 (t, $J$ = 7.5, 1H), 7.70–7.66 (t, $J$ = 7.5, 1H), 7.58–7.55 (s, 1H), 7.55–7.48 (m, 2H), 7.30–7.23 (t, $J$ = 7.9, 2H), 7.16–7.09 (d, $J$ = 7.6, 2H ), 6.90–6.82 (t, $J$ = 7.3, 1H );$^{13}$C NMR (151 MHz, $CDCl_3$)

δ 144.58, 136.92, 132.52, 131.50, 129.42, 128.11, 127.82, 127.53, 126.92, 126.90, 126.68, 125.10, 123.29, 122.48, 122.39, 120.83, 117.99, 114.77.; ESI-MS: m/z $[M+H]^+$ calculated for $C_{20}H_{16}N^+$ : 270.12773; found: 270.12791. HPLC: purity 99.9%.

***Synthesis of N-phenyltriphenylen-2-amine (PTA)*** 2-Bromotriphenylene (0.50 g, 1.62 mmol), aniline (0.23 g, 4.88 mmol), 2-dicyclohexylphosphino-2',4',6'-triisopropylbiphenyl (80 mg), sodium *tert*-butoxide (0.47 g, 4.88 mmol), and toluene (30 mL) were added to a three-necked flask. The reaction mixture was degassed by bubbling with argon for about 30 min. Subsequently, $Pd_2(dba)_3$ (0.05 g, 0.09 mmol) was added, and the reaction mixture was stirred at 110 °C under an argon atmosphere for 6 h. After cooling to room temperature, the mixture was filtered, and the solvent of the filtrate was removed by rotary evaporation. The crude product was then separated and purified by silica gel column chromatography using dichloromethane/petroleum ether (v/v=1:4) as the eluent. The resulting solid was recrystallized twice from dichloromethane/methanol under ultrasonication to give a white solid powder (0.26 g, yield 50.0%). $^1H$ NMR (600 MHz, DMSO-$d_6$) δ 8.79–8.76 (m, 1H), 8.75–8.72 (d, $J$ = 8.0 Hz, 1H ), 8.68–8.63 (t, $J$ = 8.0 Hz, 2H ), δ 8.63–8.59 (s, 1H), δ 8.54–8.50 (m, 1H), δ 8.35–8.31 (s, 1H), 7.73–7.68 (m, 2H), 7.68–7.64 (t, $J$ = 7.4 Hz, 1H), 7.62–7.58 (t, $J$ = 7.3 Hz, 1H), 7.50–7.46 (d, $J$ = 8.7 Hz, 1H), 737–7.32 (t, $J$ = 7.7 Hz, 2H), 7.30–7.25 (d, $J$ = 7.7 Hz, 2H), 6.97–6.91 (t, $J$ = 7.2 Hz, 1H); $^{13}C$ NMR (151 MHz, $CDCl_3$) δ 142.72, 142.20, 131.12, 130.20, 129.96, 129.58, 129.38, 128.73, 127.27, 127.24, 127.03, 126.17, 124.73, 124.02, 123.33, 123.30, 123.27, 122.62, 121.70, 118.64, 118.36, 110.20; ESI-MS: m/z $[M+H]^+$ calculated for $C_{24}H_{18}N^+$ : 320.14338; found: 320.14359. HPLC: purity 99.9%.

***5H-benzo[a]benzo[4,5]thieno[3,2-c]carbazole (BTCz)*** Commercially available BTCz was further purified by silica gel column chromatography using dichloromethane/petroleum ether (v/v = 1:3) as the eluent, followed by recrystallization from dichloromethane/methanol under ultrasonication prior to use. $^1H$ NMR (600 MHz, DMSO-$d_6$) δ 12.67–12.63 (s, 1H), 9.26–9.22 (d, $J$=8.4 Hz, 1H), 9.01-8.97 (d, $J$=8.3 Hz, 1H), 8.78–8.75 (d, $J$=8.1, 1.2 Hz, 1H), 8.29–8.25 (dd, $J$=7.9, 0.7 Hz, 1H), 8.25–8.21 (d, $J$=7.8 Hz, 1H), 7.89–7.84 (m, 1H), 7.83–7.80 (m, 1H), 7.79–7.76 (d, $J$=8.1 Hz, 1H), 7.70–7.66 (m, 1H), 7.56–7.52 (m, 2H), 7.45–7.41 (m, 1H). HPLC: purity 99.6%.

***4,4′-(Indolo[3,2-b]carbazole-5,11-diyl)dibenzoic acid (IbCzA)*** IbCzA was prepared according to the literature method reported by our group.[5] $^1$H NMR (600 MHz, DMSO-$d_6$) δ 13.27–13.07 (s, 1H), 8.39–8.36 (d, $J$=7.8 Hz, 2H), 8.36-8.34 (s, $J$= Hz, 2H), 8.32–8.28 (m, 4H), 7.94–7.90 (m, 4H), 7.53–7.50 (d, $J$=8.2 Hz, 2H), 7.48–7.44 (m, 2H), 7.30–7.27 (t, $J$=7.1 Hz, 2H). HPLC: purity 99.9%.

**Preparation of the melamine-formaldehyde (MF) prepolymer and resin**

A formaldehyde aqueous solution with a mass concentration of 37% (10.50 g) was added to a conical flask. Subsequently, the pH value of the solution was adjusted to 8.0~9.0 using triethanolamine, followed by the addition of melamine (7.35 g, 58.28 mmol). Finally, the mixture was stirred at 90 °C for 2.5 h to achieve the prepolymer of the MF resin. After drying at 140 °C for 12 h, 1 g of the prepolymer solution gave 0.83 g of MF polymer.

**General procedures for the preparation of the doped MF polymer films**

The PNA was dissolved in tetrahydrofuran (THF) to prepare a stock solution with a concentration of 2.00 mg/mL. Subsequently, 50, 250, 500, and 5000 μL of the stock solution were added to 1.00 g of the MF prepolymer, respectively. The resulting mixtures were treated with ultrasonic in a water bath for 10 min to promote uniform dispersion and then drop-casted onto glass slides. The samples were heated at 150 °C for 40 min to induce polymerization and cross-linking, and then a series of PNA-doped MF polymers with dopant concentrations of 0.012%, 0.06%, 0.12%, and 1.20% in the mass ratio was obtained. The doped MF polymers were ground using an agate mortar and pestle, and the resulting powders were used to prepare PNA-MF polymer films with different dopant concentrations by hot-pressing at 160 °C under a pressure of 16 MPa. Similarly, PPA-MF and PTA-MF polymer films could be successfully prepared by incorporating PPA and PTA as dopants.

**General procedures for the preparation of the doped MF polymeric microspheres**

Firstly, melamine (4.23 g, 33.54 mmol) and a formaldehyde solution (37wt% aq., 5.77 g) were added to a round bottom flask. After adjusting the pH to 8~9 using triethanolamine, the mixture was stirred at 90°C to prepare a clear and transparent solution. Subsequently, a PNA solution (5.00 mg/mL, 2.0 mL) was added, and the resulting solutions were stirred at room temperature for 10 min to yield a PNA-MF prepolymer solution. On the other hand, a PVA solution was prepared (6.00 mg/mL, 100 mL), and its pH was adjusted to 5~6 using acetic acid. The PNA-MF prepolymer was then added to the PVA solution at 60 °C, and the pH of the solution was

quickly adjusted to 5~6 using acetic acid again. The mixture was stirred at 60 °C for 30 min at a stirring rate of 500 r/min. Afterward, the resulting emulsion was centrifuged at a rate of 5000 r/min, and the solid residue was washed with deionized water four times. Finally, the product was dried in a vacuum oven at 50 °C for 7 h, and the PNA-MFs-0.12% polymeric microspheres were obtained. Similarly, the other polymeric microspheres could be successfully prepared by incorporating PPA, PTA, BTCz, BTCz/RhB, and IbCzA as dopants, respectively.

**Possible mechanism for the formation of compact and permanent 3D covalent network in the MF polymer matrix**

The compact and permanent 3D covalent network in the MF polymer matrix probably forms through a step-growth polycondensation process. Initially, under alkaline conditions, formaldehyde undergoes nucleophilic addition to the amino groups of melamine, generating hydroxymethyl (-$CH_2OH$) derivatives via methylolation. Subsequent curing drives condensation reactions between these hydroxymethyl groups and remaining amino/imino hydrogens or other hydroxymethyl groups under elevated temperature or acidic conditions. This results in the formation of covalent methylene (-$CH_2$-) and methylene ether (-$CH_2$-O-$CH_2$-) bridges connecting the melamine ring cores. Due to the high functionality of melamine, these condensation reactions propagate extensively in three dimensions, leading to the formation of a dense, rigid, and permanent 3D covalent polymer network.[6]

## II. Typical Luminescent Microspheres with Organic Afterglow Properties

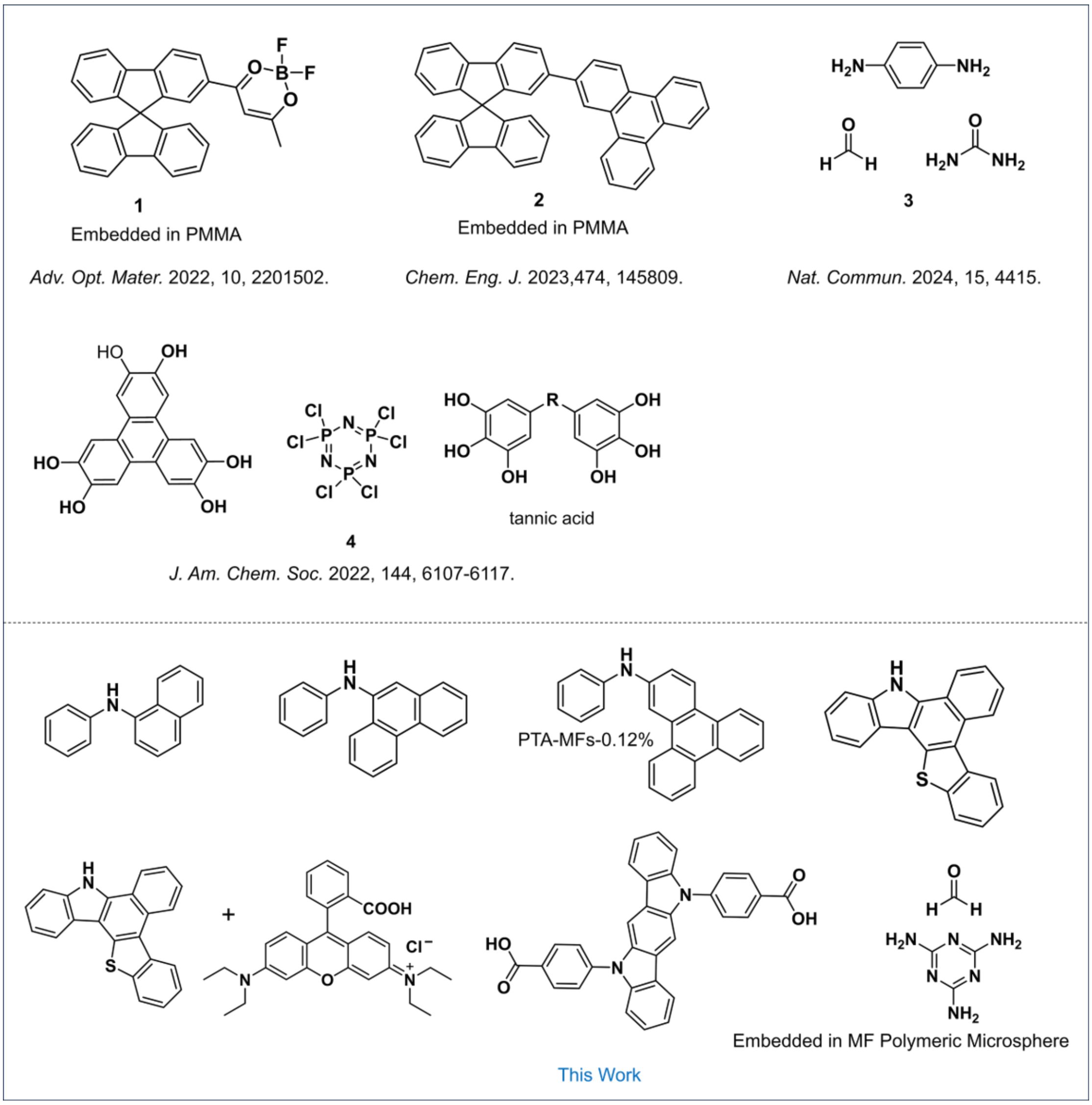


**Figure S1.** Typical luminescent microspheres with organic afterglow properties.[7]

**Table S1.** Typical luminescent microspheres with organic afterglow properties [a]

| System | Emission (nm) | $\tau_{Phos.}$ (s) | $\Phi_{phos.}$ (%) | Solvent Resistance |
|---|---|---|---|---|
| **1** | 521 | 1.53 | N/A | In Water |
| **2** | 518 | 1.7 | N/A | In Water: > 12 days |

| | | | | |
|---|---|---|---|---|
| | | | | In organic solvents: N/A |
| **3** | 451 | 0.123 | N/A | NA |
| **4** | 482 | 0.00129 | 0.07 | NA |
| **PTA-MFs-0.12%** | 525 | 1.39 | 18.0 | In Water: > 10 days<br>In organic solvents: > 10 days |

[a] In this work, the polymeric microsphere is defined as a spherical or near-spherical polymer material with a diameter in the range of 0.1~100 μm.

## III. $^{1}$H NMR, $^{13}$C NMR, and High-Resolution Mass Spectra Results

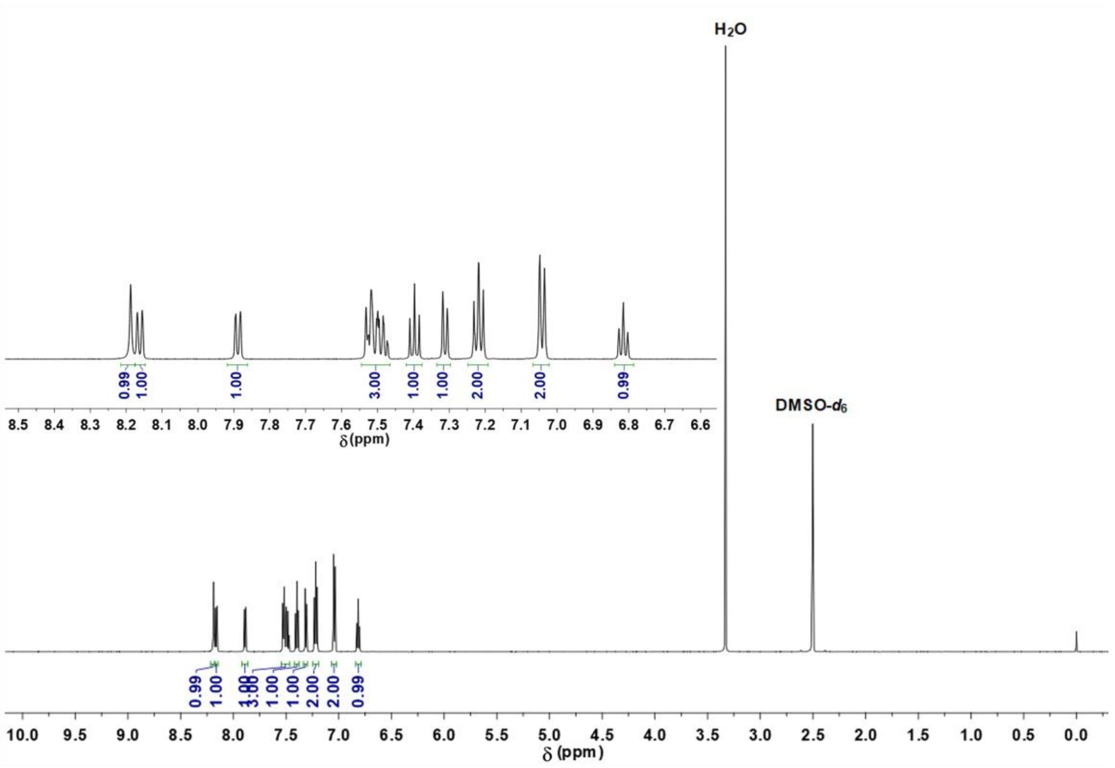


**Figure S2.** $^{1}$H NMR spectrum of PNA (in DMSO-$d_6$)

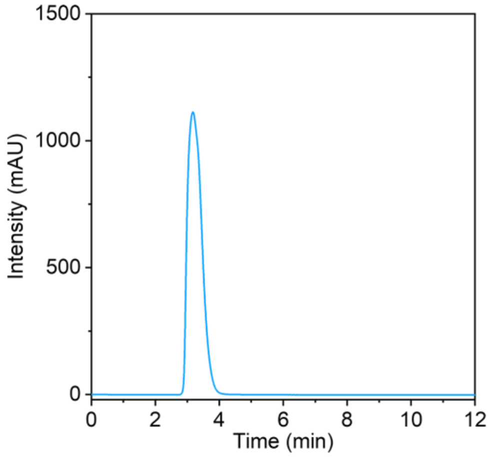


**Figure S3.** HPLC result of PNA.

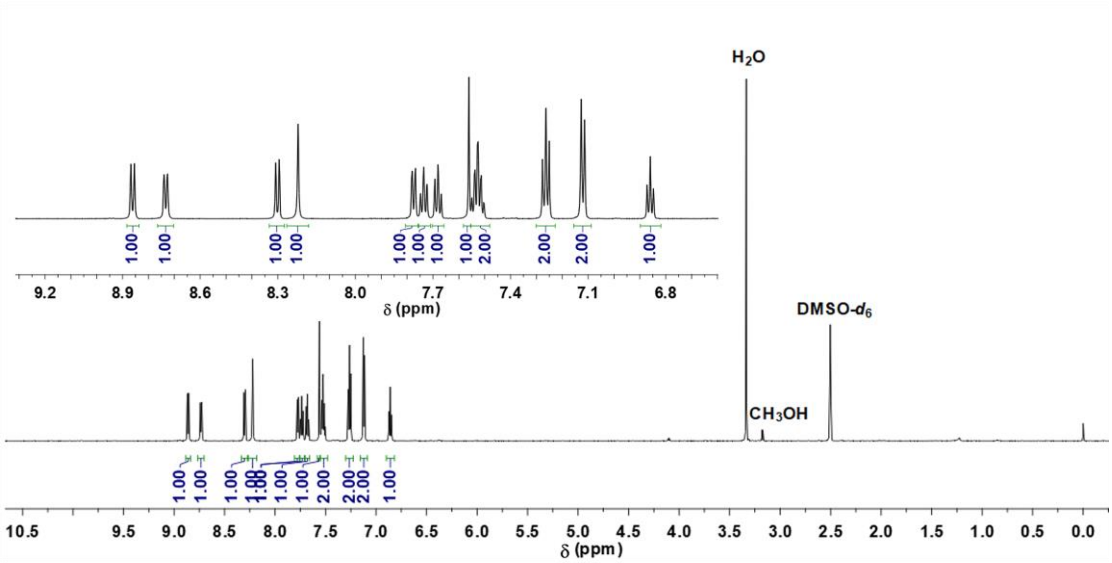


**Figure S4.** $^{1}$H NMR spectrum of PPA (in DMSO-$d_6$)

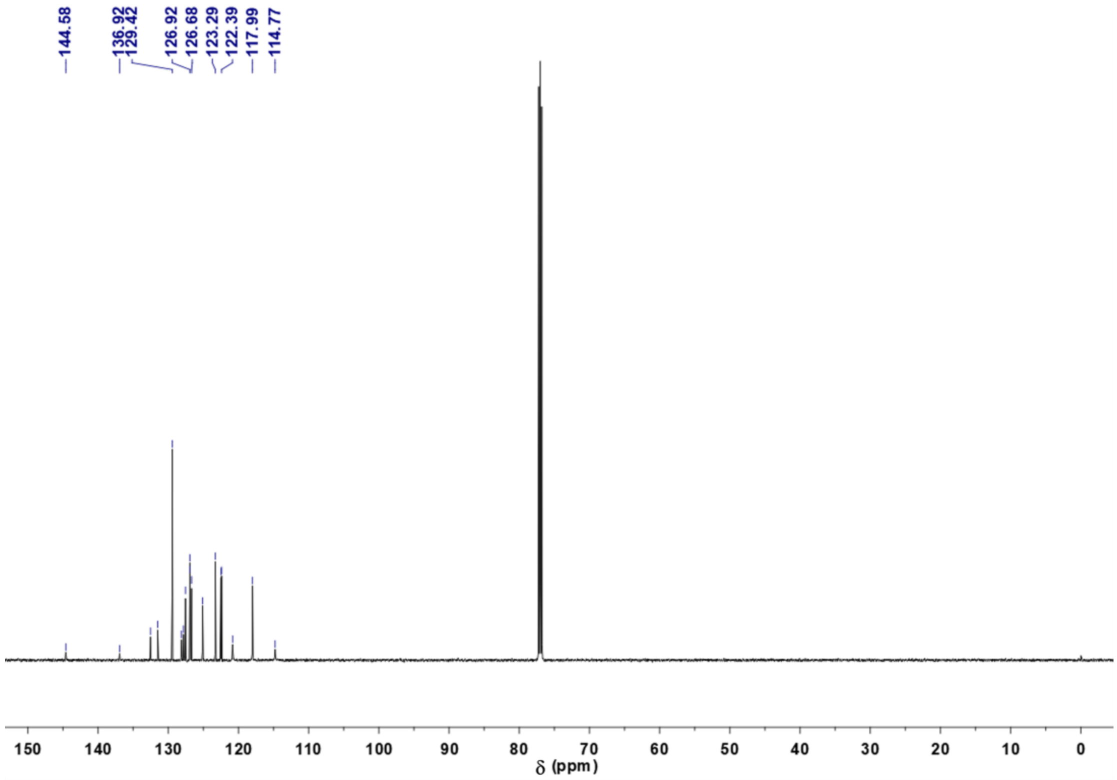


**Figure S5.** $^{13}C$ NMR spectrum of PPA (in $CDCl_3$)

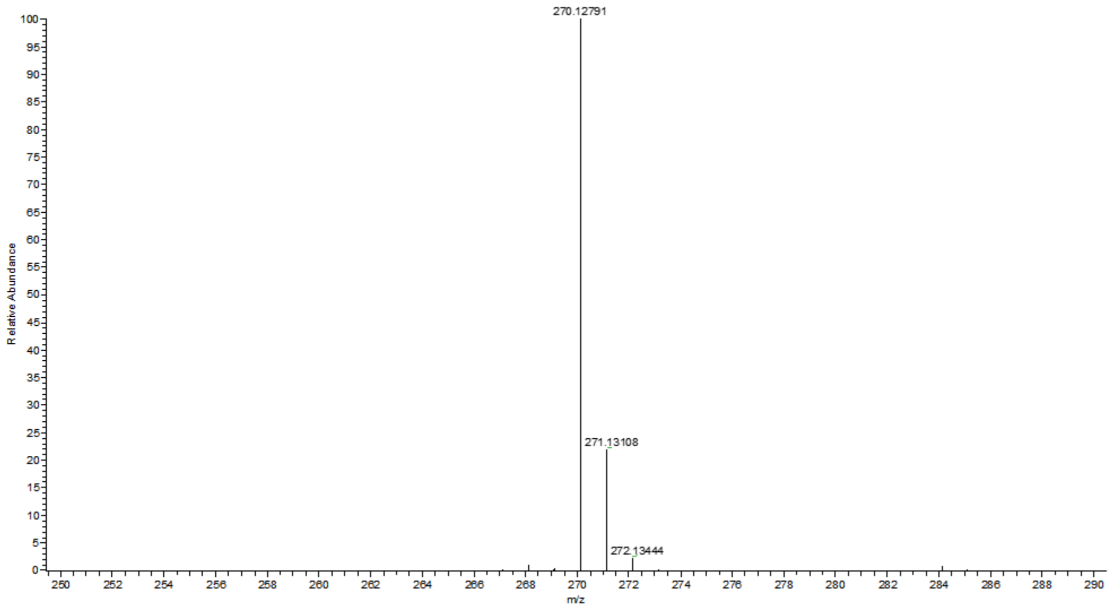


**Figure S6.** ESI-MS of PPA.

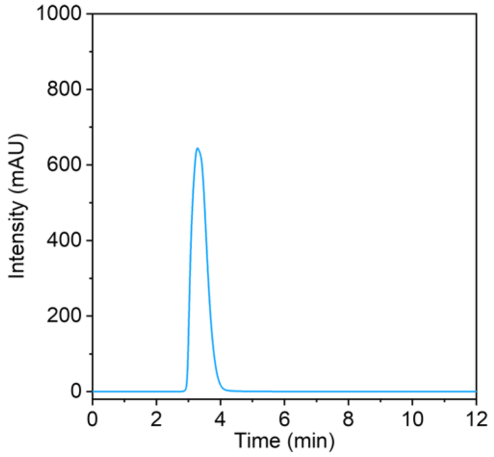


**Figure S7.** HPLC result of PPA.

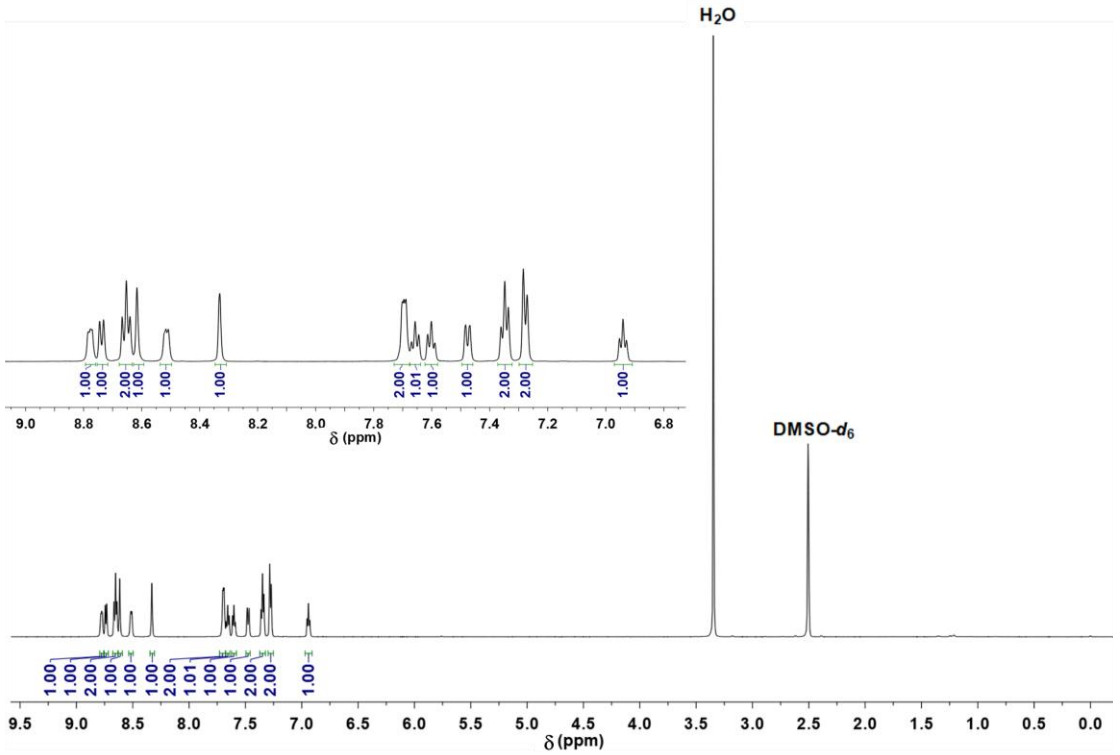


**Figure S8.** $^1$H NMR spectrum of PTA (in DMSO-$d_6$)

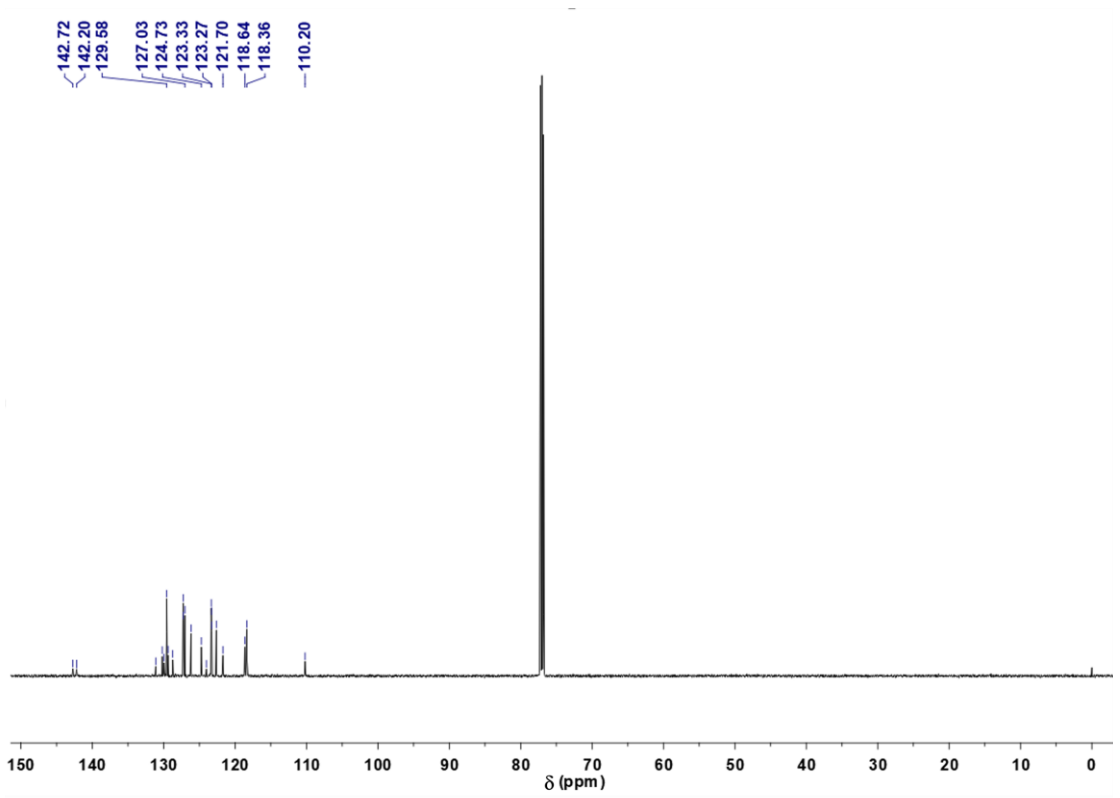


**Figure S9.** The $^{13}C$ NMR spectrum of TPA (in $CDCl_3$).

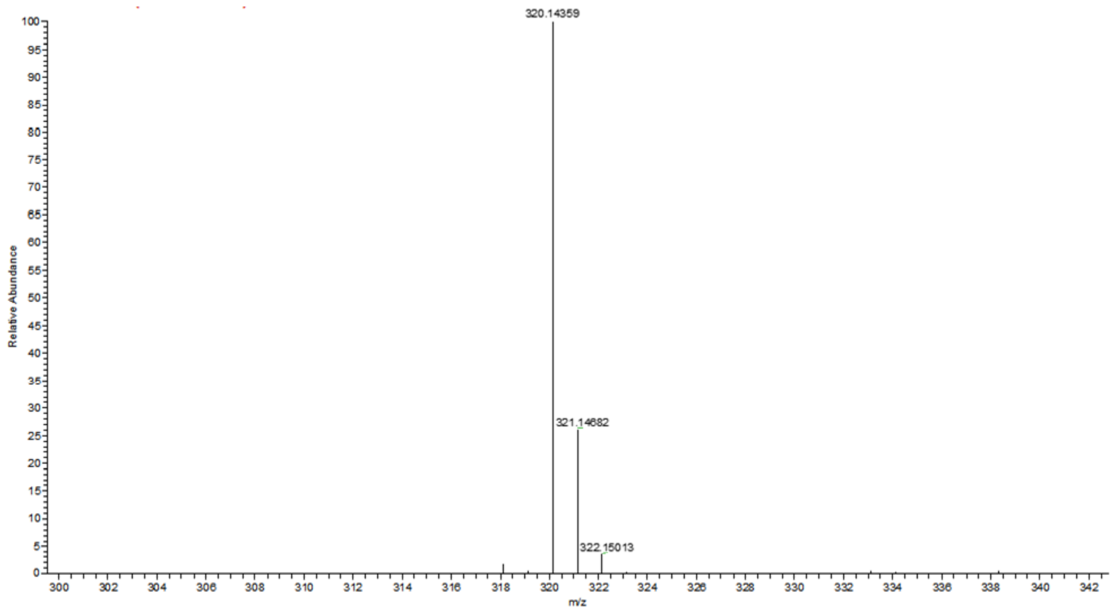


**Figure S10.** ESI-MS of TPA.

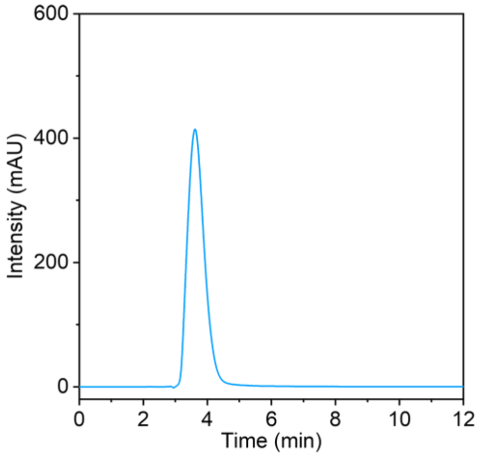


**Figure S11.** HPLC result of PTA.

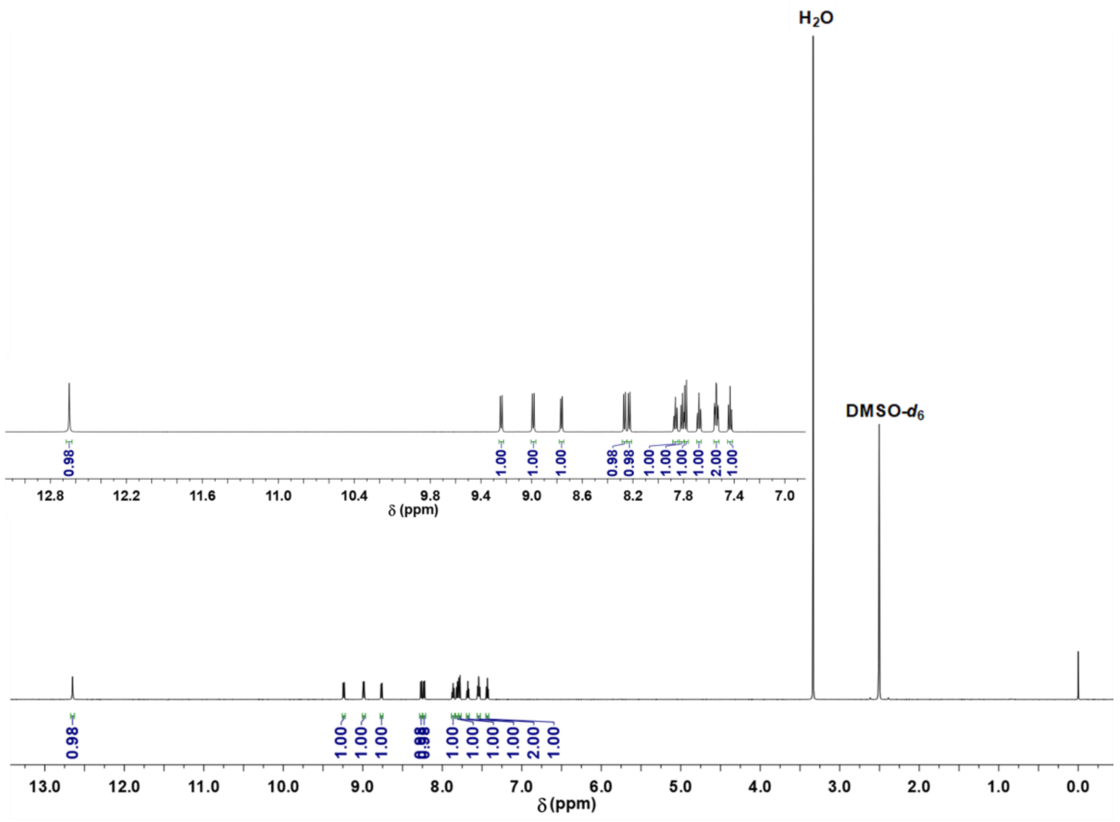


**Figure S12.** $^{1}$H NMR spectrum of BTCz (in DMSO-$d_6$)

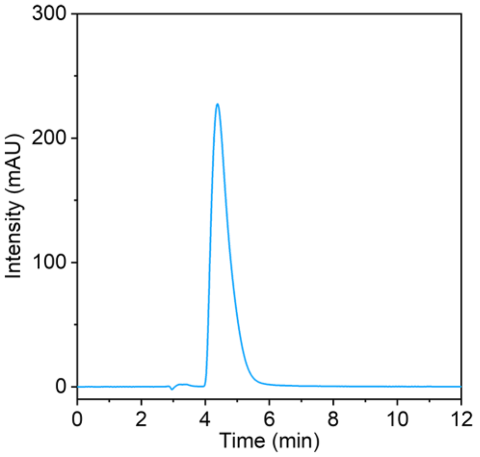


**Figure S13.** HPLC result of BTCz.

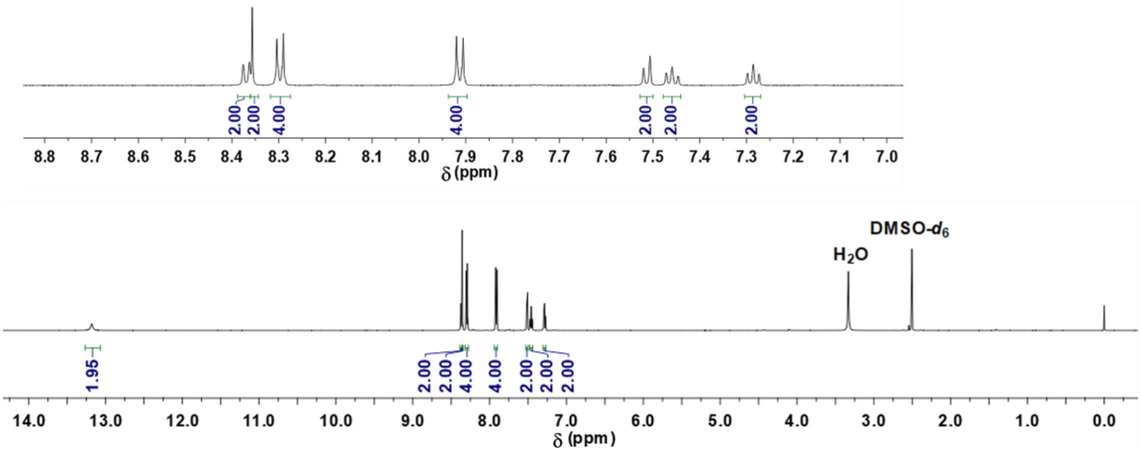


**Figure S14.** $^{1}$H NMR spectrum of IbCzA (in DMSO-$d_6$)

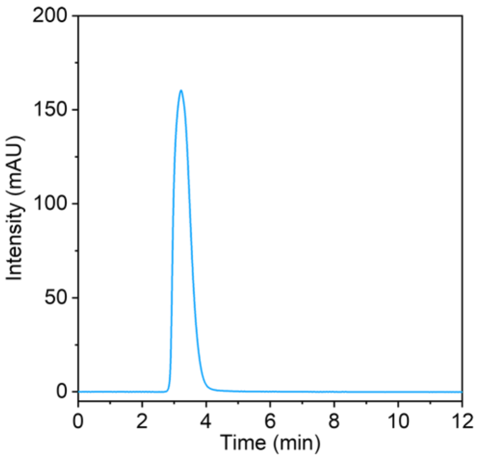


**Figure S15.** HPLC result of IbCzA.

## IV. Photophysical Properties of the Luminophores and the Doped MF Polymer Films

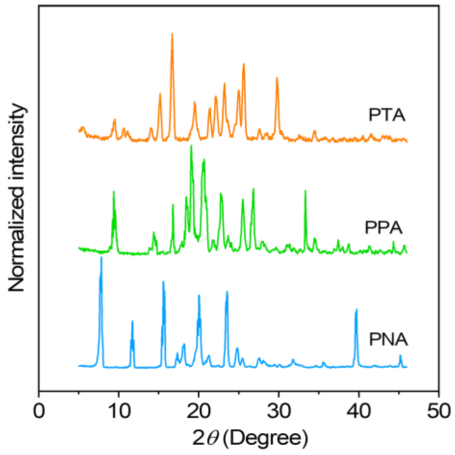


**Figure S16.** XRD patterns of the solid powders of PNA, PPA and PTA.

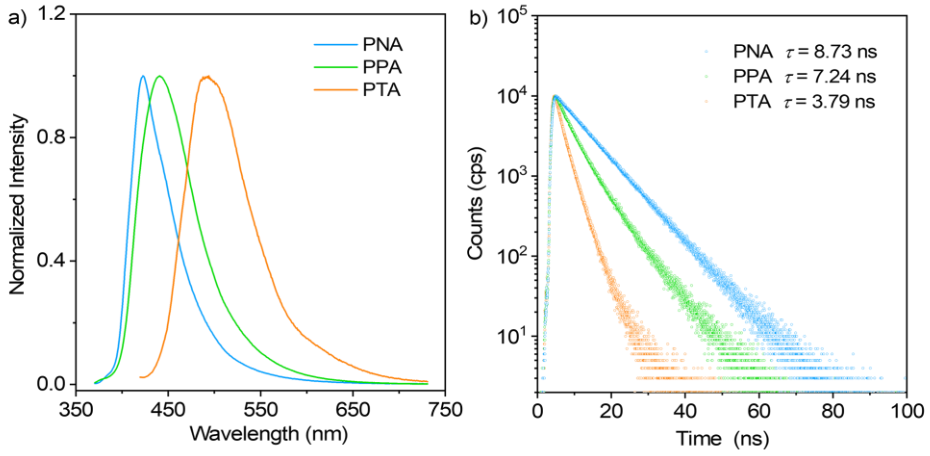


**Figure S17.** PL spectra (a) and emission decay curves (b) of the PNA, PPA, and PTA in the solid state.

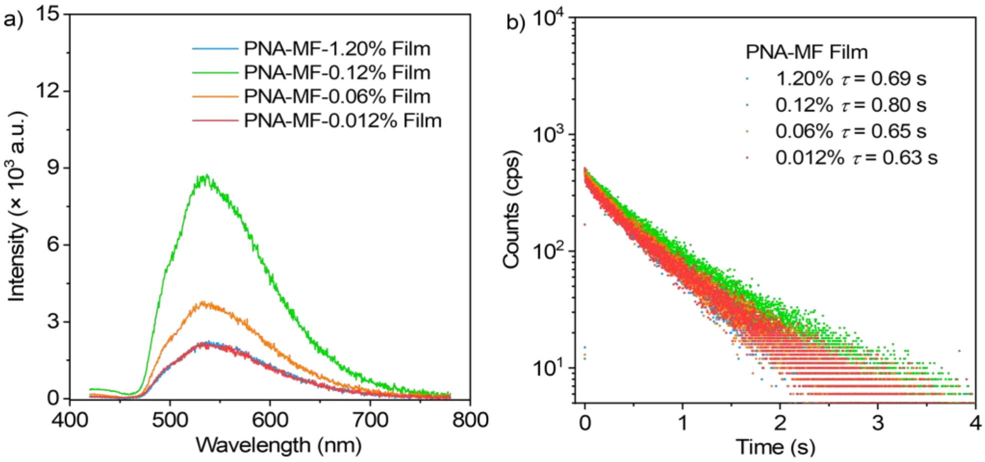


**Figure S18.** a) Delayed PL spectra of the PNA-MF polymer films with different doping concentrations under ambient conditions. b) Lifetimes of the PNA-MF polymer films at room temperature in air, monitoring at 550 nm. (Ex. 365 nm)

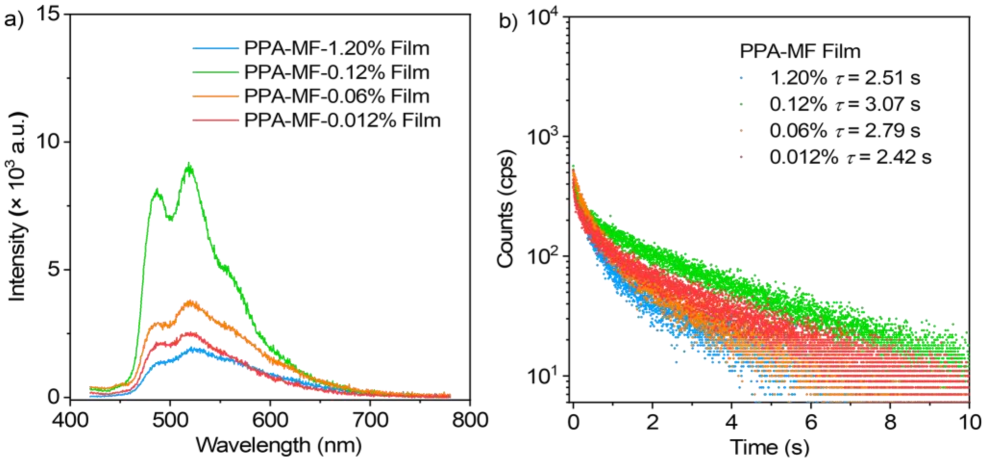


**Figure S19.** a) Delayed PL spectra of the PPA-MF polymer films with different doping concentrations under ambient conditions. b) Lifetimes of the PPA-MF polymer films at room temperature in air, monitoring at 520 nm. (Ex. 365 nm)

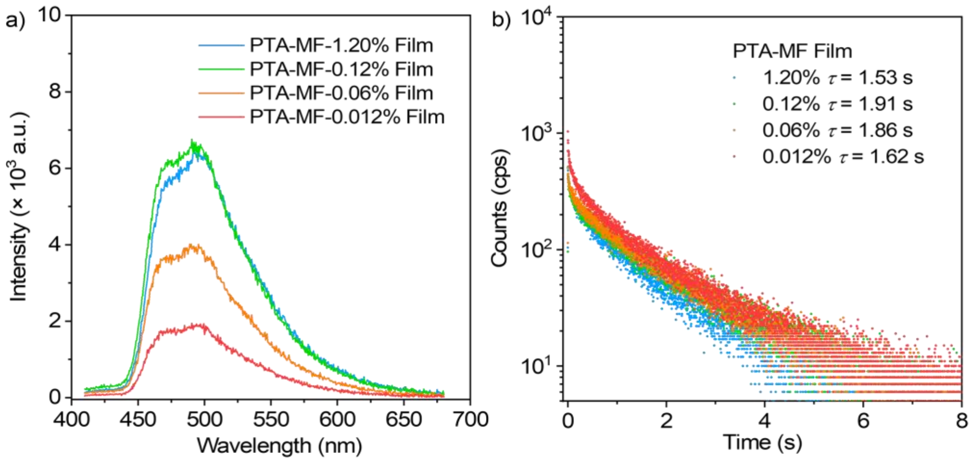


**Figure S20.** a) Delayed PL spectra of the PTA-MF polymer films with different doping concentrations under ambient conditions. b) Lifetimes of the PTA-MF polymer films at room temperature in air, monitoring at 500 nm. (Ex. 365 nm)

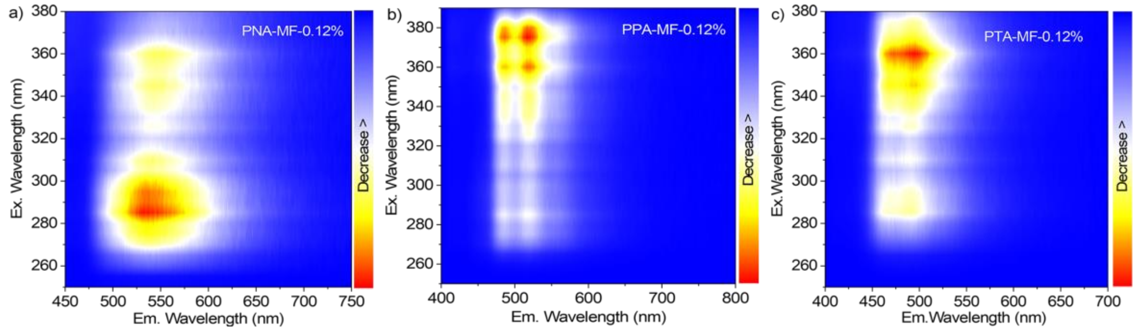


**Figure S21.** Variations of the delayed emission spectra of PNA-MF-0.12% (a), PPA-MF-0.12% (b), and PTA-MF-0.12% (c) under different excitations.

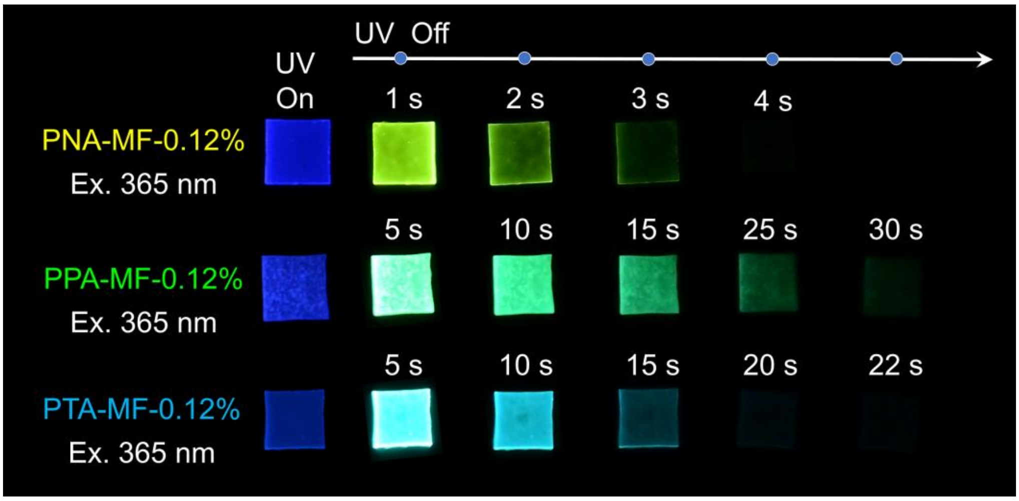


**Figure S22.** Luminescence images of the doped polymer films before and after removing UV light (light density: 30 mW/cm$^2$).

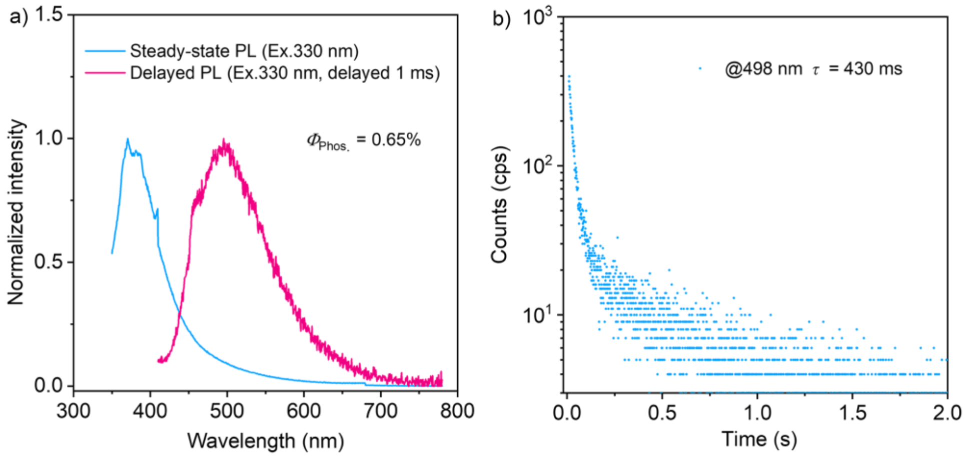


**Figure S23.** a) Steady-state and delayed emission spectra of the blank MF polymer film under ambient conditions. b) Emission decay curve of the blank MF polymer film at 498 nm under ambient conditions.

As depicted in **Figure S23**, the fluorescence and phosphorescence emission maxima of the blank MF polymer matrix are located at around 370 nm and 495 nm, respectively, which are different from those of the samples doped with PNA, PPA, BTCz, BTCz-RhB, and IbCzA. Moreover, the $\Phi_{afterglow}$ and $\tau_{afterglow}$ values of the blank MF polymer film are only 0.65% and 430 ms, respectively, indicating poor persistent luminescence performance. In this case, the phosphorescence of the MF polymer matrix has little influence on the afterglow performance of the doped samples, including those employing PTA ($\lambda_{phos.}$= 472, 495 nm; $\tau_{phos.}$=1.91 s) as the guest.

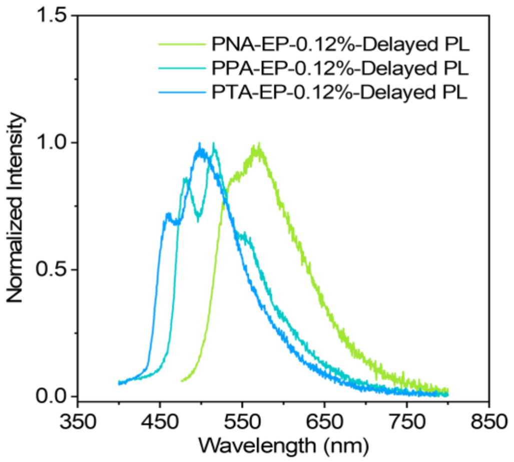


**Figure S24.** Delayed emission spectra of the PNA-EP-0.12%, PPA-EP-0.12%, and PTA-EP-0.12% films under ambient conditions.

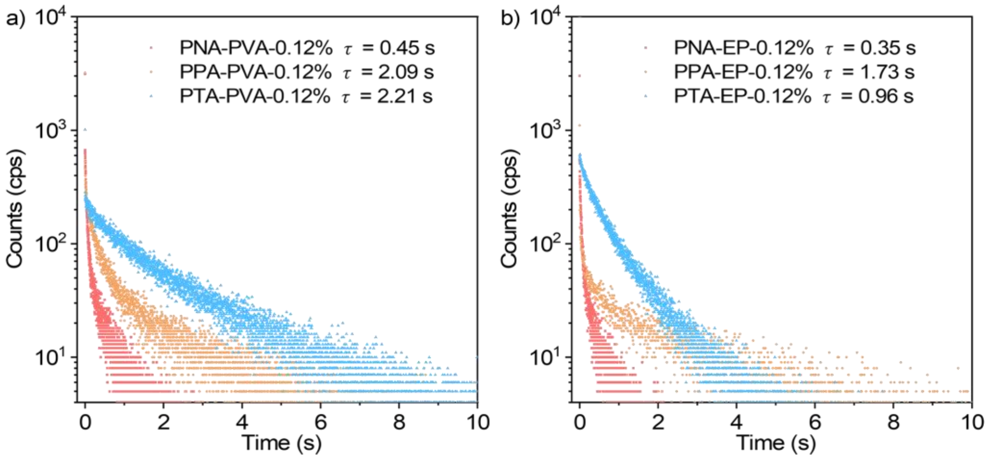


**Figure S25.** a) Emission decay curves of the PNA-PVA-0.12%, PPA-PVA-0.12% and PTA-PVA-0.12% films under ambient conditions. b) Emission decay curves of the PNA-EP-0.12%, PPA-EP-0.12% and PTA-EP-0.12% films under ambient conditions. (Ex. 330 nm)

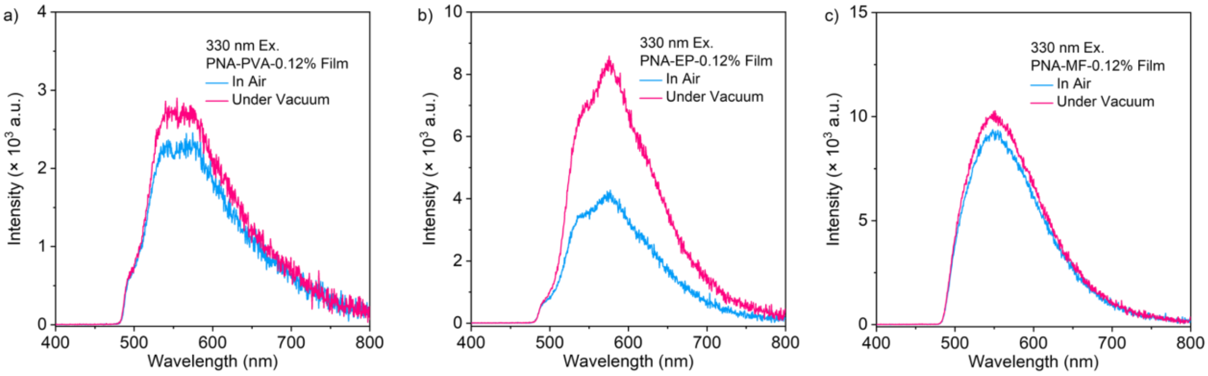


**Figure S26.** Delayed emission spectra of the PNA-PVA-0.12% (a), PNA-EP-0.12% (b), and PNA-MF-0.12% (c) films in air and under vacuum. (Ex. 330 nm)

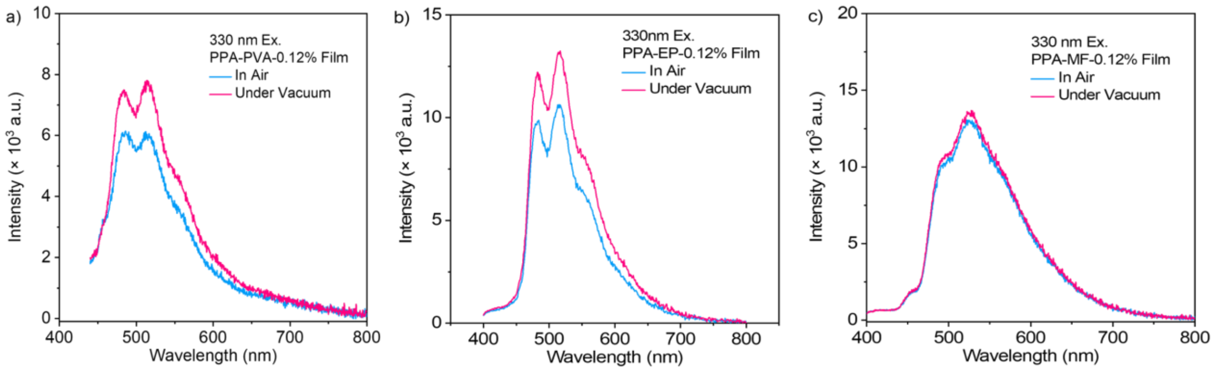


**Figure S27.** Delayed emission spectra of the PPA-PVA-0.12% (a), PPA-EP-0.12% (b), and PPA-MF-0.12% (c) films in air and under vacuum. (Ex. 330 nm)

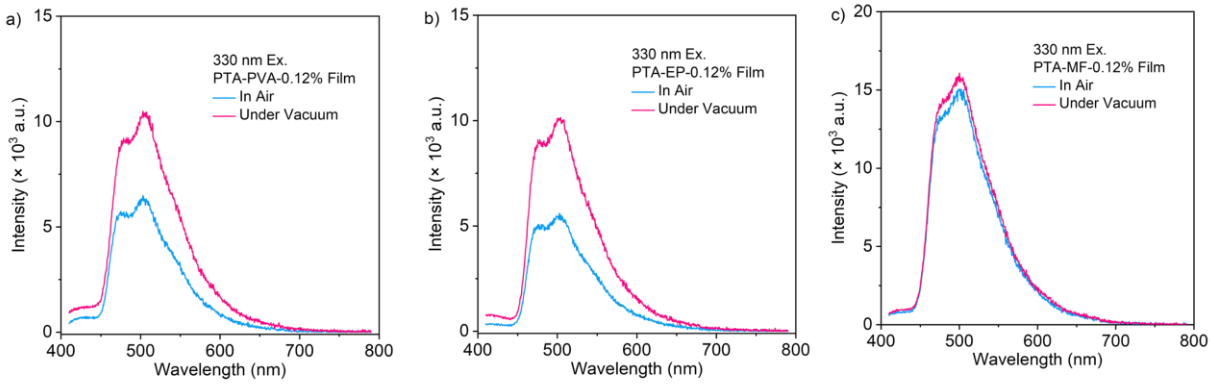


**Figure S28.** Delayed emission spectra of the PTA-PVA-0.12% (a), PTA-EP-0.12% (b), and PTA-MF-0.12% (c) films in air and under vacuum. (Ex. 330 nm)

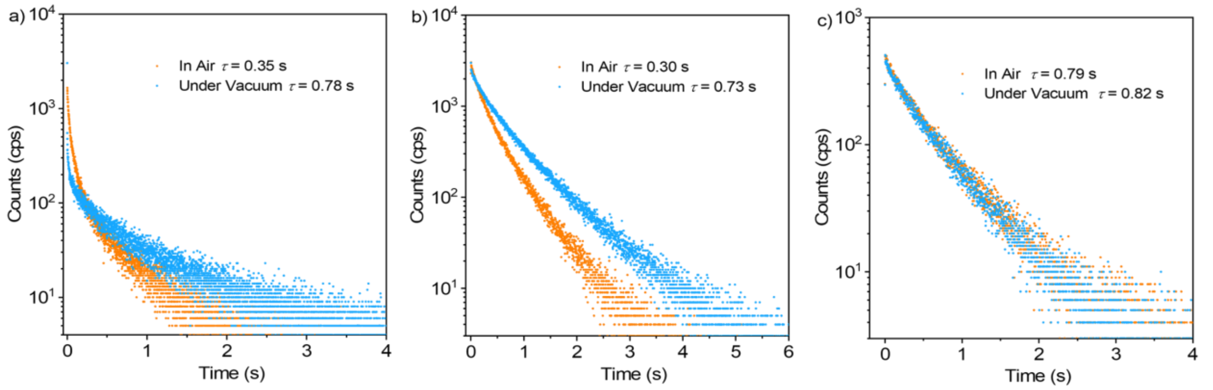


**Figure S29.** Emission decay curves of the PNA-PVA-0.12% (a), PNA-EP-0.12% (b), and PNA-MF-0.12% (c) films in air and under vacuum. (Ex. 330 nm)

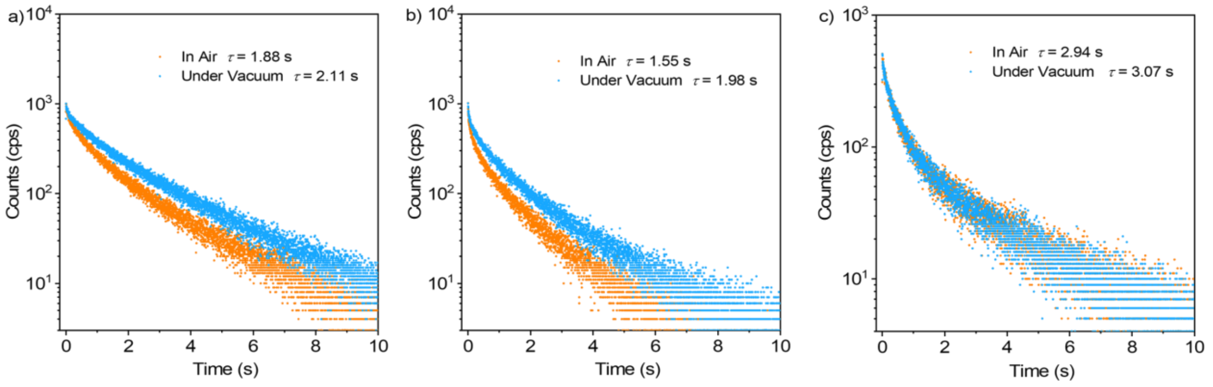


**Figure S30.** Emission decay curves of the PPA-PVA-0.12% (a), PPA-EP-0.12% (b), and PPA-MF-0.12% (c) films in air and under vacuum. (Ex. 330 nm)

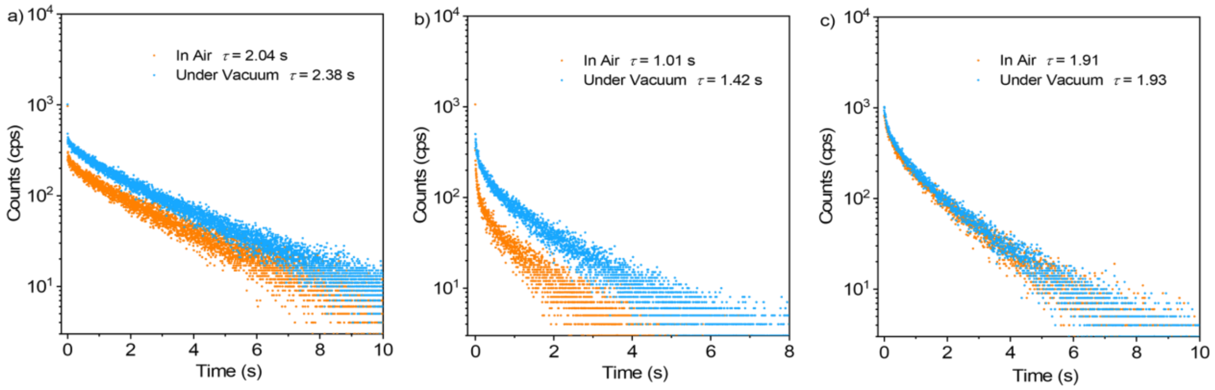


**Figure S31.** Emission decay curves of the PTA-PVA-0.12% (a), PTA-EP-0.12% (b), and PTA-MF-0.12% (c) films in air and under vacuum. (Ex. 330 nm)

## V. Photophysical Properties of the Doped MF Polymeric Microspheres

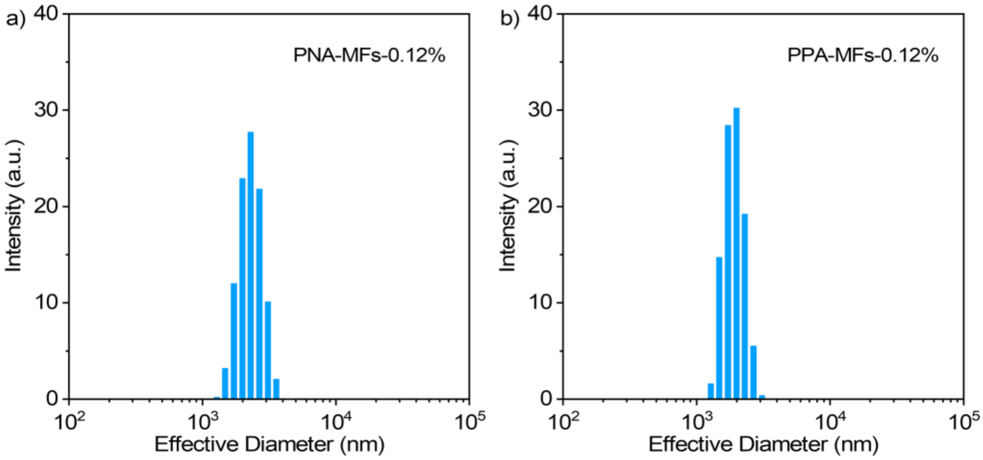


**Figure S32.** Effective diameters of the PNA-MFs-0.12% (a) and PPA-MFs-0.12% (b) microspheres dispersed in water.

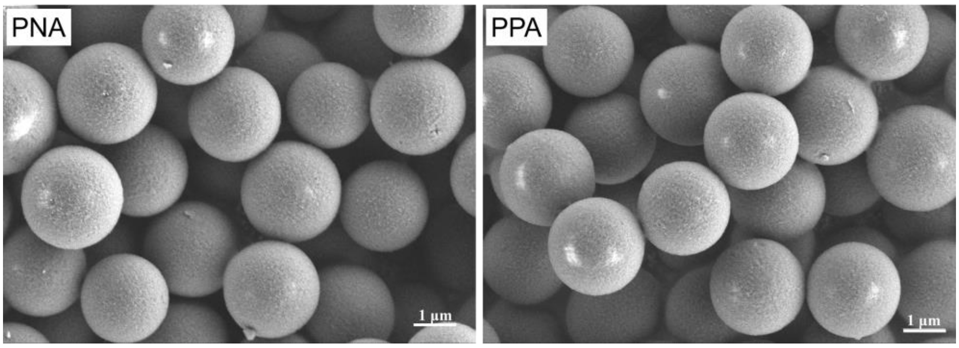


**Figure S33.** Scanning electron microscope images of the PNA-MFs-0.12% (a) and PPA-MFs-0.12% (b) microspheres under ambient conditions. (Scale bar: 1 μm)

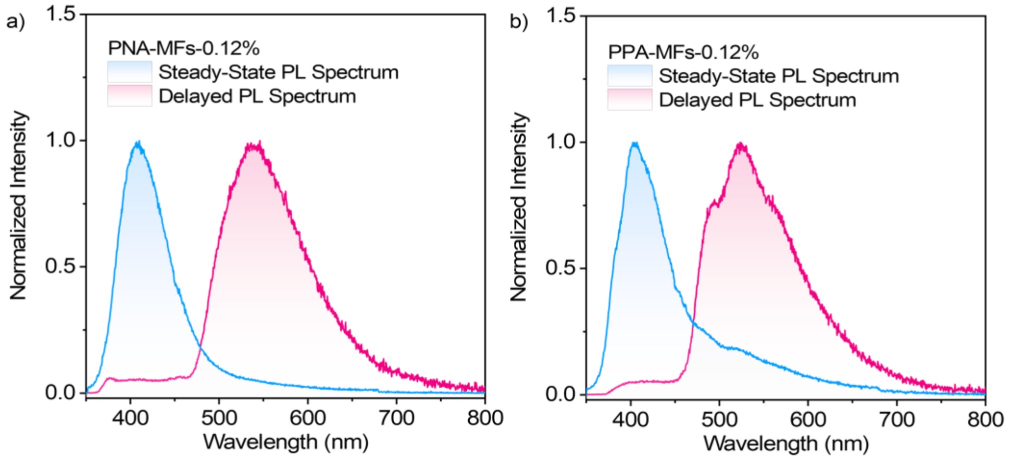


**Figure S34.** Steady-state and delayed PL spectra of the PNA-MFs-0.12% (a) and PPA-MFs-0.12% (b) microspheres under ambient conditions. (Ex. 330 nm)

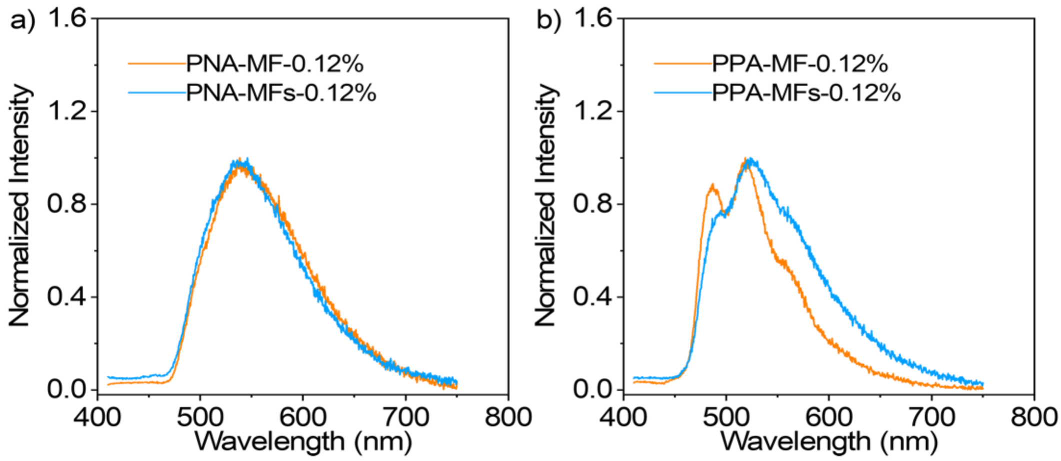


**Figure S35.** Normalized delayed PL spectra of the PNA (a) and PPA (b) in MF polymer films and microspheres under ambient conditions. (Ex. 330 nm)

The polymer-based luminescent microspheres have spherical morphologies, with effective diameters of ~2 μm. Therefore, the specific surface areas of the microspheres would be significantly larger than those of the doped MF polymer films. In this case, more guest molecules are exposed on the surfaces of the matrices, leading to the enhancement of oxygen quenching on triplet excitons and the intensification of intramolecular motions. For these reasons, the triplet excitons of the guest molecules in microspheres probably suffer from more severe non-radiative decay than those in MF polymer films, thereby resulting in a reduction in organic afterglow lifetime and quantum yield.

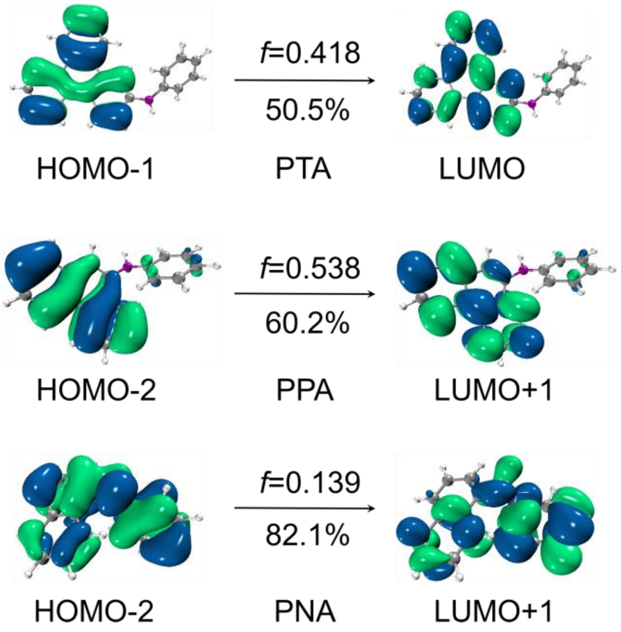


**Figure S36.** Kohn–Sham frontier orbitals of PNA, PPA, and PTA after optimization. *f* represents the oscillator strength, and the percentage is the corresponding transition probability. HOMO and LUMO are the abbreviations of highest occupied molecular orbital and lowest unoccupied molecular orbital, respectively.

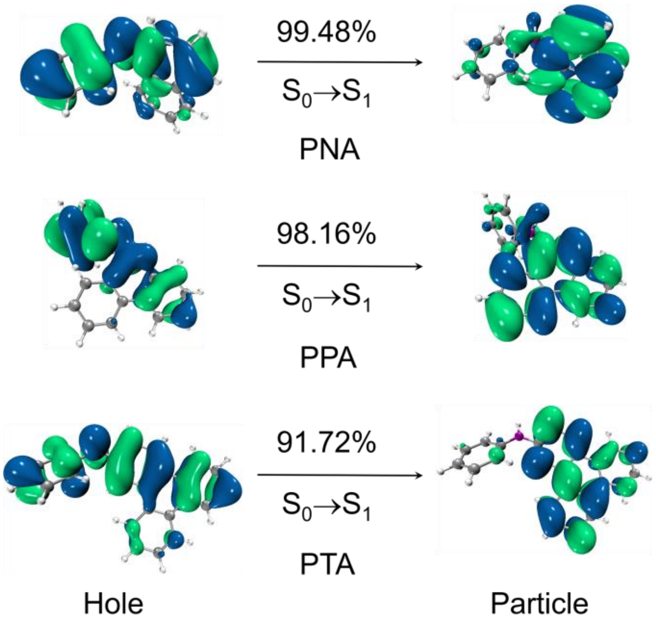


**Figure S37.** NTO characteristics of the $S_1$ states of PNA, PPA, and PTA after optimization.

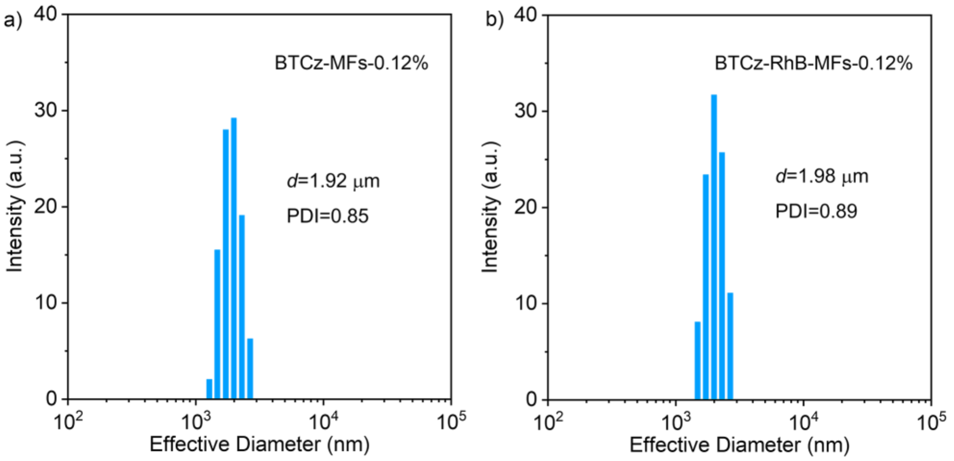


**Figure S38.** Effective diameter distribution of the BTCz-MFs-0.12% (a) and BTCz-RhB-MFs-0.12% (b) microspheres dispersed in water.

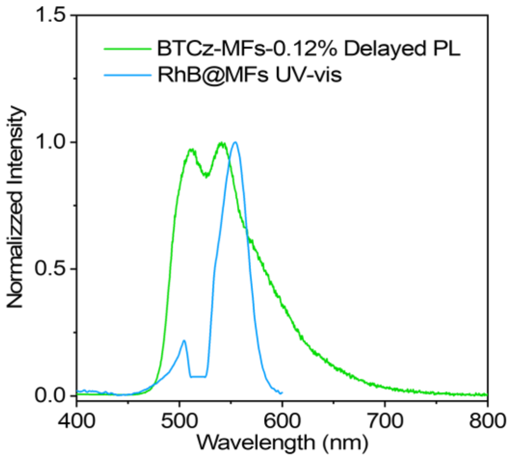


**Figure S39.** The phosphorescent emission spectrum of BTCz and UV-visible absorption spectrum of RhB in the MF polymeric microsphere under ambient conditions.

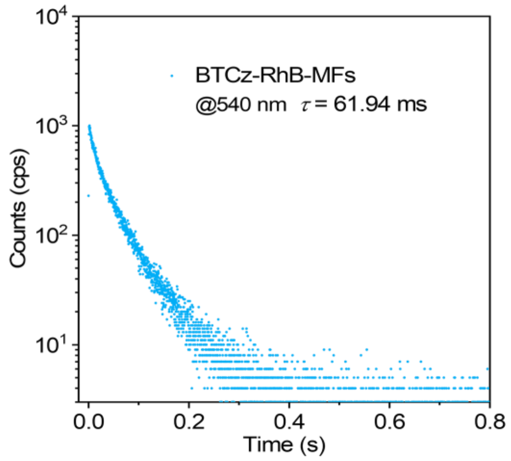


**Figure S40.** Emission decay curve and lifetime of the BTCz-RhB-MFs microspheres at 540 nm under ambient conditions.

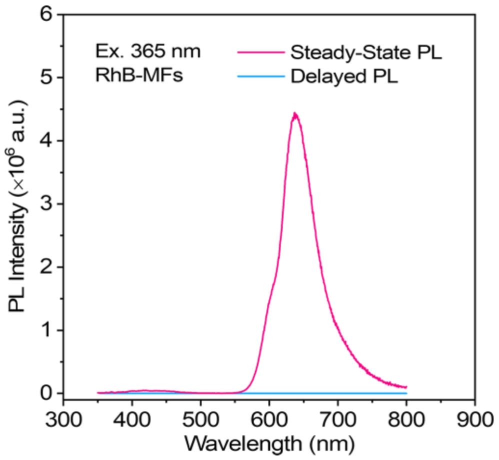


**Figure S41.** Steady-state and delayed emission spectra of the RhB-MFs microspheres under ambient conditions.

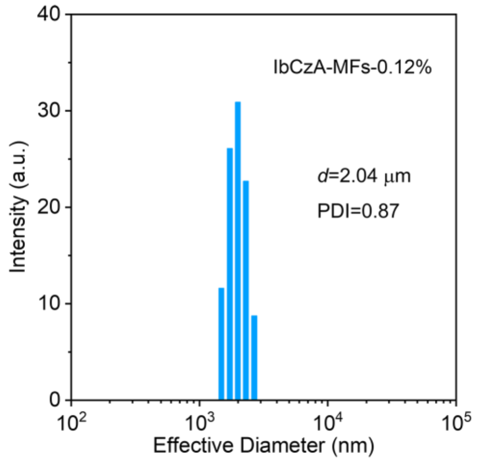


**Figure S42.** Effective diameter distribution of the IbCzA-MFs-0.12% microspheres dispersed in water.

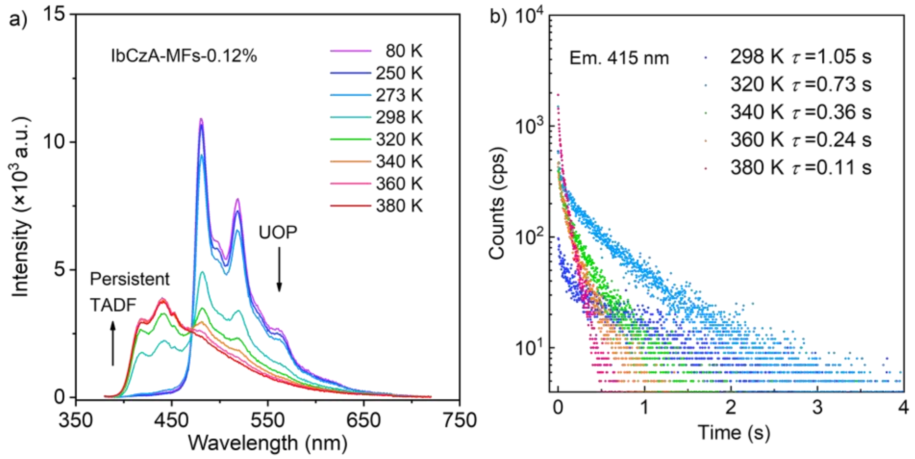


**Figure S43.** a) Delayed emission spectra of the IbCz-MFs-0.12% microspheres at different temperatures under vacuum. b) Emission decay curves and fitting lifetimes of the IbCz-MFs-0.12% microspheres at different temperatures, monitoring at 415 nm.

Temperature-dependent delayed PL spectra were measured for the IbCzA-MFs-0.12% under vacuum. The emission band at approximately 415 nm remains relatively unchanged from 80 to 273 K (**Figure S43a**). However, it gradually intensifies from 273 to 380 K, exhibiting TADF characteristics. This TADF nature is also validated by the trend in emission decays (**Figure S43b**), where the proportion of the delayed component increases significantly as the temperature increases.

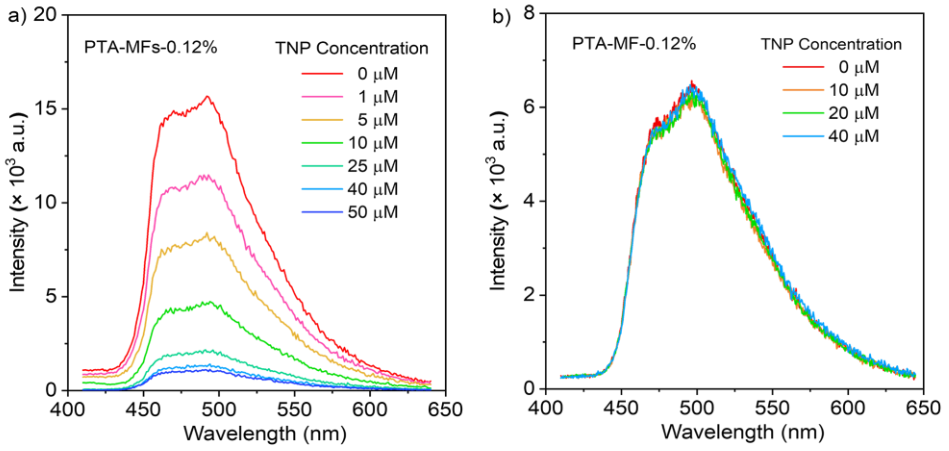


**Figure S44.** a) UOP spectra of the PTA-MFs-0.12% microspheres in water solutions with different concentrations of TNP under ambient conditions. b) UOP spectra of the PTA-MF-0.12% film after immersion in water solutions with different concentrations of TNP for 30 min under ambient conditions.

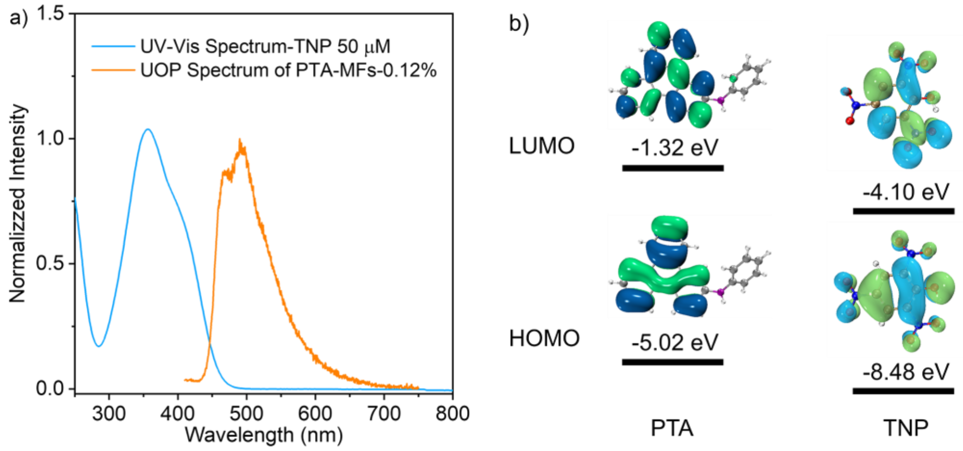


**Figure S45.** a) UV-visible absorption spectrum of TNP in water (concentration: 50 μM) and UOP spectrum of the PTA-MFs-0.12% microspheres under ambient conditions. b) The highest occupied molecular orbitals (HOMO) and lowest unoccupied molecular orbitals (LUMO) of PTA and TNP obtained by theoretical calculation.

As shown in **Figure S44**, upon exposure to a TNP solution with a concentration of 50 μM, the UOP intensity of the microspheres decreased from $1.57\times10^4$ a.u. to $1.11\times10^3$ a.u. Even in the TNP solution with a concentration of 1 μM, a significant reduction in UOP intensity was observed. These results suggest the potential of the organic afterglow microspheres for detecting traces of explosives. Given the minimal spectral overlap between the UOP emission

of PTA-MFs-0.12% and the UV-visible absorption of TNP (**Figure S45**), energy transfer from PTA to TNP molecules is unlikely to be the dominant quenching mechanism. Further investigations revealed that the LUMO level of PTA (-1.32 eV) is higher than that of TNP (-4.10 eV). Thus, the UOP quenching in PTA-MFs-0.12% is likely attributable to electron transfer from the LUMO of the guest molecules to the LUMO of TNP, followed by non-radiative deactivation back to the ground state. In contrast, TNP exhibited negligible quenching effects on the UOP of the PTA-MF-0.12% film. Even after immersion in 50 μM TNP solution for 30 minutes, no significant decrease in UOP intensity was observed. These results are presumably due to the adequate protection of guest molecules by the MF polymer matrix, which impedes contact with TNP molecules. However, the UOP intensity reduction in PTA-MFs-0.12% did not exhibit a linear relationship with increasing TNP concentration, precluding a reliable determination of the detection limit. This non-linearity likely stems from complex surface interactions between TNP molecules and the microspheres.

## Discussion

Although significant progress has been achieved for organic afterglow materials in recent years, polymer-based luminescent microspheres with organic afterglow properties are very limited. Moreover, for the sporadically reported examples, most of them show inferior persistent luminescence performance (**Figure S1 and Table S1**), which may be attributed to the relatively low ISC efficiencies of the chromophores and loose structures of the polymer matrices. Meanwhile, an undeniable fact is that polymeric microspheres exhibit a significantly larger specific surface area in comparison with macroscopic polymer materials in forms such as bulk, films, and fibers. Thus, for polymer-based organic afterglow microspheres, the probability of guest molecules locating on the material surface and contacting air is significantly increased, which remarkably weakens the restriction imposed by common polymer matrices on guest molecular motion and the protection of triplet excitons. In this case, the non-radiative deactivation of triplet excitons, caused by intramolecular motions of luminophores and the quenching effects of atmospheric oxygen, becomes extremely severe. Accordingly, the large specific surface area is also a critical reason for the unsatisfactory organic afterglow performance of common polymeric microspheres. Moreover, the reported microspheres usually emit blue to green phosphorescence, and those with satisfactory red persistent luminescence have not yet been reported to date. In addition, polyvinyl alcohol (PVA), poly(methyl methacrylate) (PMMA), polyacrylamide (PAM), polyacrylic acid (PAA), and polylactic acid

(PLA) are commonly used as matrices for the development of polymer-based organic afterglow materials in various forms and scales, including microspheres. However, they have poor resistance to water and polar organic solvents, which essentially limits their real-world applications. Consequently, it remains a formidable challenge to develop polymer-based luminescent microspheres with efficient and full-color organic afterglow under ambient conditions, especially those that can still produce conspicuous persistent luminescence in different solvents and matrices.

In this work, highly robust polymeric microspheres with efficient and full-color organic afterglow have been developed for the first time by embedding organic luminogens into the MF polymer matrices with a compact and permanent 3D covalent network via host-guest doping and emulsion polymerization. The proposed strategy enhances the overall afterglow performance of polymer microspheres by designing or selecting different luminophores with high ISC efficiency as guests, and leveraging the rigid and dense three-dimensional covalent network of MF polymers to significantly suppress intramolecular motions of guest molecules, thereby reducing non-radiative deactivation of their triplet excitons. The results show that PTA-MFs-0.12% presents a conspicuous organic afterglow with a high $\Phi_{phos.}$ of 18.0% and an ultralong $\tau_{phos.}$ of 1.39 s under ambient conditions, representing state-of-the-art comprehensive organic afterglow performance of luminescent polymeric microspheres. When compared with PVA and EP polymers, luminophores in MF polymer matrices exhibit better afterglow performance, with minor variations in afterglow intensity and lifetime under different atmospheres. These observations suggest that the MF polymer matrix likely plays a crucial promoting role in enabling the as-prepared polymer microspheres to achieve efficient UOP emission. In the meantime, the doped MF polymeric microspheres can still produce significant persistent luminescence and exhibit little change in UOP lifetimes after being soaked in water and various organic solvents for over ten days, demonstrating outstanding resistance to water and organic solvents. By employing BTCz and RhB as guest molecules, the organic afterglow color of the polymeric microsphere is successfully extended to the red region via PRET. Moreover, dual-mode organic afterglow composed of persistent TADF and UOP is also achieved from polymeric microspheres using IbCzA as the guest luminogen. This work provides a universal strategy for developing polymeric microspheres with efficient, robust, and colorful organic afterglow. It may also facilitate innovative applications of organic afterglow materials in flexible and stretchable optoelectronic devices, information security, and advanced printing technology.

## References

[1] M. J. Frisch, G. W. Trucks, H. B. Schlegel, G. E. Scuseria, M. A. Robb, J. R. Cheeseman, G. Scalmani, V. Barone, G. A. Petersson, H. Nakatsuji, X. Li, M. Caricato, A. V. Marenich, J. Bloino, B. G. Janesko, R. Gomperts, B. Mennucci, H. P. Hratchian, J. V. Ortiz, A. F. Izmaylov, J. L. Sonnenberg, Williams, F. Ding, F. Lipparini, F. Egidi, J. Goings, B. Peng, A. Petrone, T. Henderson, D. Ranasinghe, V. G. Zakrzewski, J. Gao, N. Rega, G. Zheng, W. Liang, M. Hada, M. Ehara, K. Toyota, R. Fukuda, J. Hasegawa, M. Ishida, T. Nakajima, Y. Honda, O. Kitao, H. Nakai, T. Vreven, K. Throssell, J. A. Montgomery Jr., J. E. Peralta, F. Ogliaro, M. J. Bearpark, J. J. Heyd, E. N. Brothers, K. N. Kudin, V. N. Staroverov, T. A. Keith, R. Kobayashi, J. Normand, K. Raghavachari, A. P. Rendell, J. C. Burant, S. S. Iyengar, J. Tomasi, M. Cossi, J. M. Millam, M. Klene, C. Adamo, R. Cammi, J. W. Ochterski, R. L. Martin, K. Morokuma, O. Farkas, J. B. Foresman, D. J. Fox, Gaussian 16 Rev. C.01. Wallingford, CT, **2016**.

[2] Z. An, C. Zheng, Y. Tao, R. Chen, H. Shi, T. Chen, Z. Wang, H. Li, R. Deng, X. Liu, W. Huang, *Nat. Mater.* **2015**, *14*, 685.

[3] X. Gao, S. Bai, D. Fazzi, T. Niehaus, M. Barbatti, W. Thiel, *J. Chem. Theory Comput.* **2017**, *13*, 515.

[4] a) T. Lu, F. Chen, *J. Comput. Chem.* **2012**, *33*, 580; b) W. Humphrey, A. Dalke, K. Schulten, *J. Mol. Graph. Model.* **1996**, *14*, 33.

[5] Y. Yang, Y. Liang, Y. Zheng, J. A. Li, S. Wu, H. Zhang, T. Huang, S. Luo, C. Liu, G. Shi, F. Sun, Z. Chi, B. Xu, *Angew. Chem. Int. Ed.* **2022**, *61*, e202201820.

[6] a) D. J. Merline, S. Vukusic, A. A. Abdala, *Polym. J.* 2012, 45, 413; b) A. Kandelbauer, G. Wuzella, A. Mahendran, I. Taudes, P. Widsten, *Chem. Eng. J.* 2009, 152, 556.

[7] a) J. Liu, Y. Sun, G. Wang, X. Chen, J. Li, X. Wang, Y. Zou, B. Wang, K. Zhang, *Adv. Opt. Mater.* **2022**, *10*, 2201502; b) J. Huang, X. Deng, J. Li, G. Wang, X. Li, H. Yao, C. Lei, K. Zhang, *Chem. Eng. J.* **2023**, *474*, 145809; c) W. Xu, B. Wang, S. Liu, W. Fang, Q. Jia, J. Liu, C. Bo, X. Yan, Y. Li, L. Chen, *Nat. Commun.* **2024**, *15*, 4415; d) Y. Zhang, X. Chen, J. Xu, Q. Zhang, L. Gao, Z. Wang, L. Qu, K. Wang, Y. Li, Z. Cai, Y. Zhao, C. Yang, *J. Am. Chem. Soc.* **2022**, *144*, 6107.
